# Thick or Thin? Implications of Cartilage Architecture for Osteoarthritis Risk in Sedentary Lifestyles

**DOI:** 10.3390/biomedicines13071650

**Published:** 2025-07-06

**Authors:** Eloy del Río

**Affiliations:** Independent Researcher, 11520 Cádiz, Spain; eloy.delrio@uca.es

**Keywords:** osteoarthritis phenotypes/endotypes, cartilage thickness, limiting diffusion depth, zone-dependent cartilage nutrition, nutrient influx/waste outflux, chondrocyte viability, cellular homeostasis/senescence, disuse atrophy/chondropenia, sedentary behavior, physical activity/exercise

## Abstract

Osteoarthritis (OA) is a leading cause of disability worldwide and is characterized by the gradual degradation of articular cartilage in weight-bearing joints, notably the knees and hips. However, the primary morphological and anatomical determinants of the disease onset and progression remain unclear. This narrative overview examines how variations in cartilage thickness—traditionally viewed as a biomechanical protective feature—can paradoxically compromise metabolic homeostasis during prolonged sedentary behavior. Intriguingly, compelling evidence suggests that despite its superior load-bearing capacity, thicker cartilage faces greater challenges in solute transport, a limitation further exacerbated by the formation of diffusion-resistant boundary layers at the cartilage–fluid interface during immobilization. This phenomenon restricts nutrient influx and impedes waste clearance, leading to the accumulation of catabolic byproducts in deep cartilage zones and accelerated extracellular matrix breakdown, potentially influencing OA pathogenesis. By critically synthesizing current debates on mechanical loading with emerging data on metabolic dysregulation, particularly nutrient diffusion limitations, this analysis underscores the urgent need for targeted investigation of synovial–cartilage interface dynamics and chondrocyte metabolism under low-motion conditions. This study further advocates for strategic research focusing on often-overlooked, silent metabolic imbalances among sedentary populations and recommends early-intervention strategies, such as periodic joint mobilization, ergonomic adaptations, and public-health campaigns, to reduce prolonged sitting, preserve joint function, and guide more effective prevention and management approaches for non-traumatic OA in contemporary contexts.

## 1. Introduction: Context and Overview

Osteoarthritis (OA) is a widespread multifactorial disease that affects millions of people globally [1]. This condition is increasingly emerging as a prominent cause of pain, functional limitation in daily activities, and a significant economic burden in terms of healthcare costs, primarily attributed to the need for joint replacement surgery [2]. The prevalence of OA increases with age, reaching 80 percent in individuals over 65 years of age in developed nations [3]. In the United States, diagnosed OA is the second leading cause of chronic disability after cardiovascular disease. However, the likelihood of asymptomatic early OA remains uncertain but is expected to be high, particularly in young adults [4,5,6]. This expectation is based on the increasing utilization of total knee and hip arthroplasty [7,8]. Emerging evidence suggests the existence of several endotypes of early-stage OA, each representing distinct mechanisms involved in the development of the disease [9]. While OA is currently understood as a complex disease involving not only the entire articular organ [10] but also the physiology of the whole body [11,12], its predominant hallmark is the progressive deterioration of the joint cartilage. This specialized connective tissue covering the ends of bones provides a smooth, low-friction surface that facilitates effortless movement [13]. Hyaline cartilage also acts as a shock absorber and distributes the load across the joint to shield bones from damage [14]. However, given its limited regenerative capacity, cartilage can gradually deteriorate over time, which is often attributed to factors such as ‘wear and tear’, injury, or mechanical stress [15,16,17]. Damage or loss of cartilage can lead to OA, causing pain, stiffness, and decreased mobility in affected joints [18]. Anthropological studies have shown that OA has been a widespread condition across five continents since ancient times [19,20], with evidence of the disease found in the remains of early *Homo sapiens*. Despite OA being a constant presence throughout human history, recent epidemiological data suggest an unprecedented acceleration in joint deterioration not seen in earlier periods [21]. However, the severity of this condition differs among various load-bearing joints. The prevalence of knee and hip OA is progressively increasing, particularly in developed nations with aging demographics and sedentary lifestyles [22,23]. This trend raises questions regarding the underlying factors contributing to the escalating prevalence of these joint disorders among contemporary populations.

The unique morphology of the human lower limb joints plays a crucial role in minimizing the stresses and strains that arise from repetitive high-magnitude ground and joint reaction forces during bipedal walking and running [24,25]. Unlike other mammals with similar body weights, *Homo sapiens* exhibits comparatively larger lower limb joints, such as the knee and hip joints, which have evolved to effectively support body weight and mitigate the impact of ground reaction forces during the gait cycle. This larger size not only facilitates a greater contact area between bones, but also optimizes the distribution of mechanical loads, consequently reducing the susceptibility to bone fractures [26,27]. As such, the size and structural architecture of human lower limb joints represent essential features of our locomotor system, highlighting the cartilage evolutionary adaptations that have enabled efficient bipedal locomotion [24,28]. Notably, thicker central regions of the condyles demonstrate greater resistance to cellular injury, whereas thinner hyaline cartilage is more vulnerable to fractures under lower-impact forces [28]. These adaptations were especially beneficial for enduring prolonged walking and running during hunting or for evading predators in prehistoric environments. However, in the contemporary postmodern era, when physical activity is becoming increasingly optional and less necessary for daily life [29], these evolutionary adaptations that were once advantageous may have unforeseen consequences. As sedentary lifestyles become increasingly prevalent, gaining a comprehensive understanding of the implications of the morphological features of articular cartilage is crucial for addressing the potential challenges associated with the altered demands of the human musculoskeletal system. Therefore, exploring the intricate interplay between cartilage thickness and modern lifestyle choices is imperative for clarifying their impact on the health and function of freely movable synovial joints (diarthroses) and for efficiently managing the challenges of preserving cartilage health in an increasingly sedentary population.

It is well-established that the sedentary lifestyle prevalent in modern human populations has led to a significant increase in the incidence of coronary heart disease, type 2 diabetes, and overweight/obesity, yet the negative impact of this lifestyle on joint health has received disproportionately little attention. Recent research has suggested that physical inactivity and sedentary behavior may be significant risk factors for developing OA [4,5,21,30]. In the new millennium, sedentary lifestyles are common worldwide, with people spending increasing amounts of time sitting or engaging in low levels of physical activity [29]. As previously highlighted, this shift has been associated with a significant increase in the prevalence of OA, notably affecting the knee and hip joints. Despite the clear association between sedentary behavior and OA [5], the specific etiopathogenic mechanisms by which it contributes to the disease remain poorly understood. One potential factor that has received limited consideration in the literature is the limiting depth of joint cartilage, which may play a significant role in the pathogenesis of OA. Limiting depth refers to the concept that the diffusion properties of synovial fluid restrict the penetration of nutrients into the deeper layers of the cartilage, resulting in nutritional stress and metabolic imbalance [31,32,33,34,35]. Unlike vascularized tissues such as muscles, synovium, or bone—where cells lie within approximately 0.1 mm of a capillary—chondrocytes reside within pericellular matrices (chondrons) in avascular cartilage, where diffusion distances may extend to several millimeters or even centimeters [32]. An important factor underlying the limiting-depth concept is the presence of stagnant liquid films or unstirred layers adjacent to the cartilage surface, which act as diffusion barriers and may critically modulate bioactive solute transport from the synovial fluid into deeper cartilage zones. Hyaline cartilage relies on passive molecular diffusion from the joint surface via synovial fluid as its primary source of nourishment [36,37]. In this context, the synovium can be conceptualized as a physiological interface analogous to a “placenta” for avascular cartilage, leveraging synovial fluid as the primary vehicle for delivering essential nutrients, growth factors, and signaling molecules across the diffusion barriers necessary to maintain chondrocyte viability and matrix integrity. Accordingly, joint cartilage poses a significant challenge: as depth increases, nutrient availability progressively decreases owing to consumption by the overlying cells [31,32,34,35]. This limitation in nutrient supply may elevate the risk of OA, resulting in persistent concerns throughout adulthood. Although the pathophysiological mechanisms are not yet fully understood, nutrient deprivation can lead to metabolic imbalances in the deeper layers of articular cartilage. Consequently, this disruption of cartilage cell homeostasis may not only trigger catabolic degradation of the extracellular matrix (ECM) but also impair the necessary anabolic activity of chondrocytes, thereby contributing to the development of OA. This may play a significant role in the pathogenesis of cartilage degradation. Although physical inactivity is likely to contribute to various mechanisms, it has been hypothesized that sedentary behavior could exacerbate this inherent vulnerability. As mentioned earlier, this study further examined the influence of modern sedentary behavior on this vulnerability, which can be attributed to the accumulation of stagnant fluid pools on the cartilage surface during prolonged or chronic periods of inactivity. This often-overlooked surface phenomenon can result in nutrient deprivation of tissue cells and contribute to a higher occurrence of early-stage OA among younger individuals. By understanding the mechanisms underlying this trend, we can develop strategies to mitigate its impact and improve cartilage health outcomes for future generations. The implications of this study, in terms of critical insights into OA as both a multifaceted medical condition and a significant social challenge, are discussed, underscoring the need for integrated clinical and public health strategies.

## 2. Objectives, Research Design, and Methodology

This narrative review aims to elucidate how variations in hyaline cartilage thickness influence OA onset and progression under prolonged sedentary conditions, an inactive lifestyle, or immobilization by integrating biomechanical, anatomical, morphological, and metabolic data. Specifically, this study aims to (1) delineate the mechanistic interplay between cartilage structure, nutrient transport limitations, and OA risk in sedentary populations; (2) provide a critical synthesis of the current empirical evidence; and (3) propose testable mechanistic models alongside methodological strategies to guide future research. Structured searches of PubMed/MEDLINE, Embase, Web of Science, and Scopus were systematically performed for English-language studies published between January 1950 and May 2025 using combinations of keywords such as “joint/articular/hyaline cartilage”, “cartilage thickness”, “solute transport”, “passive diffusion”, “sedentary lifestyle/behavior”, “physical inactivity”, “immobilization”, “osteoarthritis phenotypes/endotypes”, “chondrocyte viability”, or “disuse atrophy”. These searches were supplemented by reference list harvesting of seminal works on joint cartilage structure, transport physiology, mechanical modeling, and cellular organization, as well as noteworthy contributions identified from congress abstracts. Eligible studies included original peer-reviewed articles that quantitatively measured cartilage thickness (e.g., via MRI or histology), characterized solute transport dynamics or unstirred layer phenomena, evaluated chondrocyte metabolic responses under static or unloading conditions, or correlated cartilage architecture with OA biomarkers. Priority was given to studies closely aligned with the scope of this review, particularly those presenting robust evidence, well-defined research questions, and comprehensive analytical approaches. Obsolete, repetitive, and conceptually unrelated studies were excluded. Non-peer-reviewed commentaries, case reports, traumatic/post-surgical models, and non-English publications were also excluded from the study. Data were extracted in a standardized form capturing the study design, measurement techniques, diffusion coefficients, metabolic endpoints, and principal findings. Although no formal risk of bias assessment was conducted, methodological heterogeneity was critically appraised, and findings were synthesized thematically—integrating classical diffusion theory with in vivo and in vitro studies into a unifying “sedentary–thickness axis” framework—to define key mechanistic pathways and highlight critical gaps for future research into preventing cartilage thickness-mediated OA in inactive populations.

## 3. Rethinking Alternative Perspectives on Osteoarthritis: Challenging the ‘Wear and Tear’ Paradigm and Exploring the ‘Use It or Lose It’ Theory

The relationship between post-traumatic and idiopathic OA remains uncertain based on the available evidence [38,39]. The prevailing paradigm for idiopathic OA, which assumes similarities to post-traumatic OA in terms of pathomechanics, poses a significant challenge. This standard paradigm suggests that OA is primarily a “procatabolic disease” resulting from mechanical factors, such as repetitive joint use or excessive loading, which leads to gradual ‘wear and tear’ of the articular cartilage over time [15,17,40,41]. Although this conventional perspective recognizes the cumulative influence of biomechanical stress on joint health and emphasizes the significance of managing and reducing joint overload to prevent the onset of OA, it fails to explain several observations related to the etiology of idiopathic disease. Notably, it does not account for the substantial disparity in OA prevalence between the talocrural (ankle) joint and the tibiofemoral (knee) and coxofemoral (hip) joints [42,43], the higher prevalence of OA in females compared to males specifically in the latter joints [44,45], inconsistent correlations with impact and loading activities such as running [46,47] and other weight-bearing sports [48], increased prevalence in sedentary human populations [22,29], and the apparent resistance of free-ranging arboreal primates to the disease [49,50]. Although excessive mechanical loading is a well-recognized factor that may predispose synovial joints to OA, the increased risk associated with abnormal or excessive loading is not universal. Therefore, a thorough examination of alternative paradigms is essential for a deeper understanding of nontraumatic OA, considering these clinical observations and recognizing that factors beyond mechanical stress are integral to the development of the disease.

**Figure 1:**
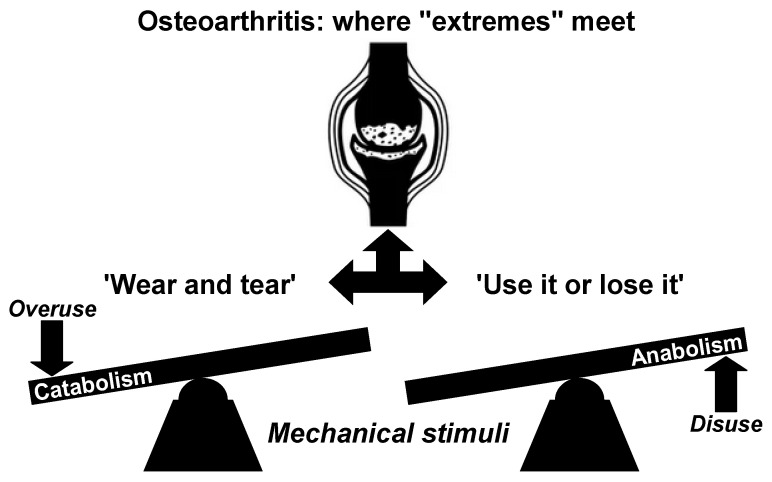
Conceptual model illustrating two mechanobiological pathways—“wear-and-tear (overuse)” and “use-it-or-lose-it (disuse)”—that trigger ultra-early metabolic imbalance in hyaline cartilage, leading to OA. The left panel depicts how repetitive mechanical overload leads to cumulative matrix microdamage, enzymatic degradation, and progressive loss of cartilage integrity, whereas the right panel shows how insufficient mechanical stimulation during sedentary behavior or immobilization disrupts anabolic pathways by impairing bioactive solute transport, compromising chondrocyte homeostasis, and weakening the tissue structure. A central OA joint icon emphasizes the convergent pathological outcomes of both pathways. Exploring both theoretical perspectives and their association with underlying causes provides a comprehensive understanding of the multifaceted nature of metabolic imbalances in hyaline cartilage and their contribution to early-stage OA pathogenesis.

The ‘wear and tear’ (or mechanical stress) theory has predominantly derived from laboratory animal studies focusing on post-traumatic OA [16,28,51,52,53,54,55,56], leading to the assumption that idiopathic disease follows a similar pattern and is primarily a consequence of mechanical joint destruction [57]. However, it is important to acknowledge that the pathological changes observed in animal studies represent an advanced stage of OA and do not provide a comprehensive model for explaining all forms of the disease. In early OA, biochemical changes suggest a gradual and subtle process of cartilage deterioration, characterized by altered synthesis within the ECM, rather than mere destruction [58]. This challenges the conventional understanding of nontraumatic OA as a purely procatabolic mechanism that focuses on tissue destruction. For that reason, it is essential to explore the alternative concept of an antianabolic factor, as schematically illustrated in Figure 1, in order to enhance our comprehension of this condition. Current understanding recognizes that OA is influenced by a complex interplay of biomechanical and biochemical factors [39]. However, insufficient attention has been paid to the potential dominance of anabolic deficiencies over procatabolism in the early stages of OA, as discussed previously. In this initial phase, compromised anabolic functions within otherwise normal chondrocytes may hinder the effective restoration of physiological catabolic processes, thereby contributing to the development of OA. This interpretation suggests that nontraumatic OA can be more effectively explained as a manifestation of decreased cellular function in “sedentary joints”. Hence, the perception that OA is solely a result of mechanical factors or ‘wear and tear’ that lead to cartilage destruction is inadequate. Disregarding the antianabolic component, particularly in post-traumatic OA, may partially explain some cases of late-stage disease, but fail to fully elucidate the complexities associated with early idiopathic OA. Considering the significant increase in sedentary lifestyles, it is crucial to move beyond the simple ‘wear-and-tear’ perspective.

In light of evolving research, OA should be recognized as a multifactorial and heterogeneous condition, resulting from diverse and individualized pathways, rather than a singular, uniform process, which necessitates personalized approaches for both research and clinical practice. The widely accepted belief that OA is solely caused by ‘wear and tear’ due to loading stress on the primary weight-bearing human joints is overly simplistic and misleading. As stated in reference [38], this perspective has had a *stultifying effect on medical opinion regarding prevention and treatment of OA*. Based on the previous discussion, OA should therefore be conceptualized as a common end stage rather than a common final pathway, reflecting that while the disease manifests similarly in its advanced form, its development can arise from a variety of unique etiological factors, distinct underlying processes, and specific subtypes, including post-traumatic, metabolic, post-menopausal, and age-associated phenotypes [59,60,61]. Although the conventional ‘wear and tear’ theory adequately explains post-traumatic OA, it fails to fully elucidate the underlying mechanisms of the more common idiopathic form of the disease in the present-day understanding. In the context of nontraumatic OA, the principle of ‘use it or lose it’ (or ‘move it or lose it’) could emerge as an alternative paradigm in the era of widespread sedentarism, emphasizing the detrimental effects of joint disuse [4,5,30,51,54,62,63,64,65,66,67,68,69]. This perspective highlights the importance of maintaining regular physical activity and joint mobility in preserving the health and integrity of cartilage in weight-bearing joints. While both the conventional ‘wear and tear’ model and the often-overlooked ‘use it or lose it’ perspective contribute to our understanding of the risk factors influencing joint health and the pathogenesis of OA, adopting the latter approach may provide valuable insights into idiopathic OA. This promising approach has the potential to significantly enhance the development of more effective prevention and treatment strategies for knee and hip joints [70], especially within the context of modern, highly sedentary Western societies.

## 4. Sedentary Behavior and Osteoarthritis: A Growing Epidemic in Developed Countries

Despite significant advances in recent years, many critical aspects of the OA puzzle remain elusive, unresolved, or poorly understood. Epidemiological studies, although extensive, have not fully elucidated the underlying mechanisms that drive this condition. It is clear, however, that OA is a multifactorial disease influenced by a combination of factors such as aging, sex, genetic predisposition, joint biomechanics, metabolic imbalances, and environmental and lifestyle factors [4,5]. The increasing prevalence of OA is strongly correlated with the global rise in sedentary lifestyles [22], which have reached epidemic levels in many developed regions, including the United States and Western Europe [29]. In these regions, which are characterized by a high prevalence of sedentary behavior, OA has reached unprecedented levels [22]. In contrast, regions characterized by low rates of physical inactivity, often found in economically disadvantaged areas, demonstrate a correspondingly lower prevalence of OA. For instance, Uganda, with a sedentary population of only around 5%, exhibits a remarkably low prevalence of OA compared to the most affluent regions worldwide [22]. The prevalence of sedentary behavior, characterized by a lack of physical activity and prolonged periods of sitting or inactivity, has emerged as a major public health concern in developed nations. In the United States, over 80% of the population fails to meet the physical activity recommendations established by the World Health Organization (WHO) [71]. Unfortunately, this percentage has recently increased to nearly 90%, reaching the highest recorded levels [72]. This escalation can be attributed to reduced physical activity levels (PALs) resulting from the widespread incorporation of mechanized work, household machines, and vehicle engines into daily life, leading to a dramatic decline in activities of daily living (ADLs) [29]. The advent of the digital revolution has exacerbated this situation, with a potentially more significant impact due to the prolonged hours of sedentary lifestyles. This projection may be further exacerbated by post-COVID-19 teleworking trends, the ascent of artificial intelligence, and anticipated advancements in robotics, factors contributing to increased physical inactivity and potentially intensifying the incidence of OA. In the context of this study, this emerging phenomenon could be characterized as sedentary-associated OA or introduced as a terminological amalgamation, denoted here as *sedenthrosis*.

The emergence of combustion vehicles in recent decades has undeniably impacted the levels of physical activity and sedentary lifestyles among contemporary humans, particularly with regard to transportation. According to current estimates, the total number of registered motor vehicles worldwide is roughly 2.2 billion, and this figure is expected to double by 2040 [73]. The United States has the highest number of registered vehicles (280 million), followed by Western Europe (210 million). In these regions, there are 832 vehicles per 1000 inhabitants and 606, respectively, while in Africa, the number is only 39 per 1000 inhabitants [74]. The significant increase in vehicle ownership has been a major contributing factor to the widespread increase in sedentary behavior observed in modern industrialized societies. This is attributed not only to the extended periods of sitting during motorized journeys but also to the subsequent reduction in options for engaging in regular and active human movement [29]. Sedentary behavior is primarily associated with a seated posture during motorized transportation activities, such as driving short trips to and from work, and this trend has become increasingly common since the latter half of the 20th century. Logically, the prevalent sedentary behavior is inherently accompanied by a decrease in opportunities for physical activity, resulting in a significant reduction in the daily quantity of active joint motion [29,72]. With the widespread production and accessibility of transportation vehicles over time, passive sitting during automobile travel has emerged as a dominant form of sedentary behavior in industrialized populations. Although walking has been the primary mode of transportation throughout most human history, it has largely been replaced by automobiles in modern times. This shift towards motorized transportation has led to an increase in sedentary behavior, particularly during activities such as commuting to work.

Analyzing state-level data on commuting by bicycle and walking in the United States can provide insights into the relationship between sedentary behavior and OA. State-level data show that regions with high rates of bicycle and walking commuting are mainly concentrated in the west, whereas the south has the lowest rates of walking and bicycling to work [75]. Even with warm weather and flat terrain, these modes of transportation are less popular in the central deep southern region than in the rest of the country. While several risk factors for OA have been identified, sedentary behavior has emerged as a potentially substantial contributor to the development of the disease [4,5,21], particularly in the context of the United States. Interestingly, this association may be partly explained by the higher incidence of OA in the southeastern quadrant of the country [76], where elevated levels of sedentary behavior prevail [77]. There appears to be a strong association between sedentary behavior and OA, prompting the introduction of the novel term *sedenthrosis* to describe this specific correlation, which may alternatively be coined as *sedenthritis*—a proposed new OA phenotype linked to the degenerative effects on joint cartilage arising from modern sedentary lifestyles. In the context of this study, the proposed term *sedenthrosis*, as presented here, provides a potential correlation for various phenomena observed in established OA, including secondary ossification, tidemark reduplication, and overgrowth of other joint components [78,79,80,81,82,83,84], potentially exhibiting a higher prevalence among highly sedentary adult populations. Therefore, it is reasonable to deduce that *sedenthrosis* may correspond to an accelerated manifestation of idiopathic OA in postmodern humans. If this is correct, then the cartilage of our synovial joints could not be designed for our current chronic sedentary lifestyle. However, it does not seem that a sedentary lifestyle affects all joints of the lower limbs in the same manner. Undoubtedly, this is a piece of the OA puzzle that still requires further explanation.

## 5. Which Weight-Bearing Joints Are Primarily Affected by Osteoarthritis? The Good, the Bad and the Ugly

The question of why some joints are disproportionately affected by OA, both in terms of frequency and severity, has long been a subject of interest to rheumatologists [40]. As noted earlier, the onset of this joint disease is influenced by a variety of factors, including age, genetics, injury, and obesity. However, certain diarthrodial joints exhibit higher susceptibility to OA because of variations in their anatomy and biomechanical characteristics. Epidemiological data indicate that the ankle, knee, and hip joints, which are the main load-bearing joints of the lower limbs, exhibit different degeneration patterns. While knee and hip OA are common, idiopathic OA of the ankle is rare, with degenerative changes occurring five times more frequently in the knee (76%) than in the ankle (14%) by the age of 70 [42,43]. Although ankle OA is less frequent than that of the knee or hip (the “bad” and the “ugly”, respectively), it is mainly caused by post-traumatic OA resulting from injuries or fractures [43,85,86,87], while idiopathic OA is the dominant etiology of knee and hip OA [88,89]. The etiology of ankle OA is not well understood, but it appears probable that it has a distinct pheno-endotype compared to knee and hip OA [9]. Symptomatic ankle OA is uncommon in older individuals and occurs in less than 1% of cases in the absence of traumatic injuries [87]. Despite being subjected to significant biomechanical stress, the underlying reason for the ankle joint being resistant to OA remains an unresolved puzzle, earning it the distinction of being the “good” joint. This enigma continues to elude researchers and presents a formidable challenge to their efforts. Unraveling the intricate protective mechanisms that safeguard the ankle joint from nontraumatic OA continues to be an ongoing pursuit in the field, fueling the need for a comprehensive understanding of this fascinating resilience.

## 6. The Role of Mechanical Stress in Joint Health: Addressing Misconceptions and Complexities

**Figure 2:**
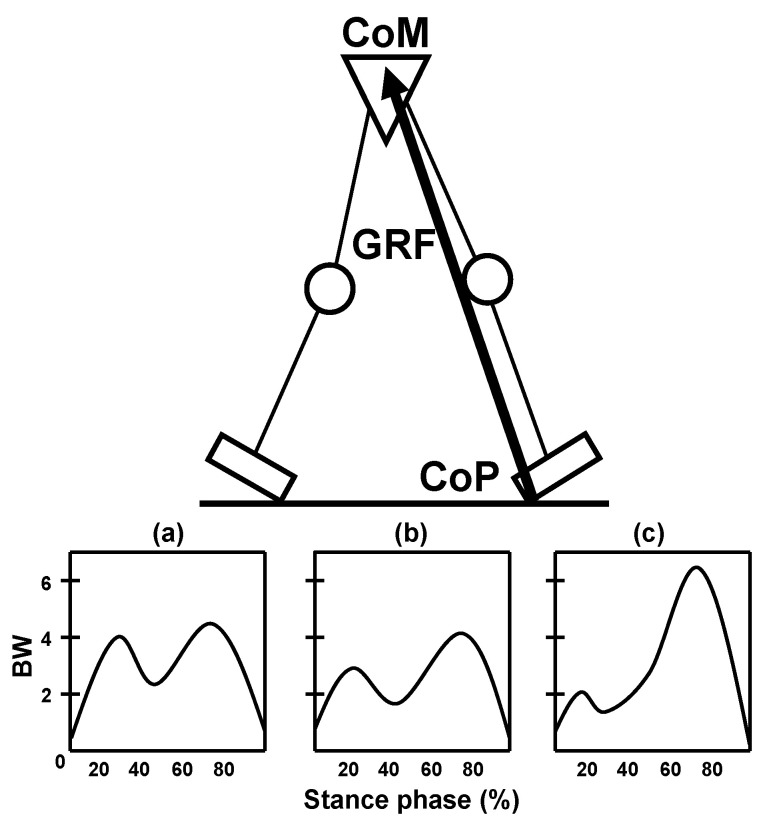
Integrated schematic and ensemble joint-contact-force profiles of the hip, knee, and ankle during the stance phase of gait. Top: Simplified stance-leg illustration showing the center of pressure (CoP), ground reaction force (GRF), and center of mass (CoM). The hip, knee, and ankle joint centers are marked by a triangle, circle, and square, respectively. Bottom: Mean normalized joint-contact forces (body weight, BW) plotted against 0–100% of the stance phase. The hip (**a**) and knee (**b**) both follow a biphasic loading pattern, showing an initial peak at load acceptance and a secondary peak at the push-off. The ankle (**c**) exhibits a modest initial peak, followed by a dominant mid-stance peak. Data sourced from [92,93].

The knees, hips, and ankles are three of the major weight-bearing joints in the human body responsible for supporting locomotion and enduring the demands of daily activities. These joints undergo a remarkable number of load cycles throughout their lives, estimated to be up to one million cycles per year [90]. Their shared function lies in the efficient transmission of high loads while minimizing friction, thereby enabling smooth and coordinated movement [13]. Mechanical stress, a fundamental aspect of cartilage physiology, plays a critical role in maintaining joint health [91]. It is defined as the force applied per unit area and directly influences the integrity and functionality of articular cartilage, synovial fluid, and other joint structures. During the gait cycle, particularly in the context of normal walking, the knees, hips, and ankles experience varying degrees of mechanical stress, as illustrated in Figure 2. On average, the knee joint experiences loading equivalent to approximately three times the body weight, the hip joint up to four times, and the ankle joint up to five times the body weight during routine weight-bearing activities [92,93]. It is intriguing that, despite experiencing the highest compression force, the ankle joint consistently demonstrates a lower susceptibility to degenerative changes in its joint structures, in contrast to the knee and hip joints, which are subjected to relatively lower levels of stress.

The size of the weight-bearing areas is a critical factor in determining the distribution of mechanical forces across joint surfaces [94]. A larger weight-bearing area enables a more efficient load distribution, reducing the stress per unit area and minimizing the risk of excessive loading on specific regions of the cartilage. Conversely, a smaller weight-bearing area concentrates the forces over a smaller surface, leading to higher stress concentrations and potentially increasing the risk of tissue damage. In humans, the weight-bearing areas of the knee [95], hip [96], and ankle [97] joints, which account for around one-third of the total articular surface area of the joint cartilage, are estimated to be approximately 1120 mm^2^, 1100 mm^2^, and 350 mm^2^, respectively. These values correspond to the regions where forces and loads are transmitted between the joint surfaces during weight-bearing activities. Notably, the contact surface in the knee and hip joints is roughly three times larger than that in the ankle joint. Thus, the pressure exerted on the cartilage of the ankle joint can be considerably higher, reaching up to three times the magnitude of pressure experienced by the other joints in the lower limbs. During walking, articular cartilage is subjected to average pressures of approximately 2 MPa in the knees, 3 MPa in the hips, and 5 MPa in the ankles, with the latter even reaching 7 MPa [92,93,95,96,97]. These magnitudes of pressure result in spatially averaged contact stress equivalent to approximately 3 times and 5 times the body weight, highlighting the significant mechanical loads endured by these three joints during normal walking [92,93]. For instance, in an individual weighing 70 kg, the load (or force per contact area) on the articular cartilage is 172, 183, and 858 N/cm^2^ in the knee, hip, and ankle joints, respectively. Although mechanical stress is a critical factor in joint health, it cannot be the only explanatory variable for the ankle joint, as it transmits relatively high forces through relatively small contact areas. Despite receiving greater compressive forces per surface unit, owing to its reduced contact area [95,96,97], the ankle shows a unique deviation from the trend observed in the development of OA. If mechanical stress does not explain the low prevalence of OA in the human ankle joint, what does? What factors make the ankle unique? How can this paradox be explained? It is evident that a theory focusing solely on biomechanical factors is inadequate for providing satisfactory answers to these questions.

## 7. The Exceptional Resilience of the Human Ankle Joint Against Osteoarthritis: Understanding the Puzzle

Mechanical factors play a significant role in the development of OA, yet the detailed mechanisms involved remain unclear. As noted previously, an intriguing aspect of this condition lies in the ankle joint, which experiences considerably higher levels of mechanical stress than the knee and hip joints during daily activities (see Figure 2). However, the ankle joint exhibits surprising resilience against the onset of non-traumatic OA. This can be attributed to various biomechanical, metabolic, and anatomical differences between the ankle and femoral joints [92,93,98,99,100,101,102,103,104,105,106,107,108,109,110]. Specifically, preservation of tensile strength in the ankle cartilage may contribute to the resilience of the joint against degeneration [98]. Moreover, this hyaline tissue demonstrates greater resistance to low-grade inflammation compared to knee and hip cartilage [101,103,105]. Chondrocytes, which are responsible for maintaining the ECM of cartilage, play a vital role through anabolic (synthesis) and catabolic (degradation) pathways. Proteoglycans, as key components of the ECM, contribute significantly to its structural integrity and function, providing resistance to compressive forces within the cartilage [111,112,113,114,115]. In contrast to other extracellular matrices, hyaline cartilage demonstrates a significant increase in the elastic modulus with higher strain rates [116]. This adaptive response enhances its capacity to resist impact damage under mechanical loading conditions, a phenomenon attributed to the high concentration of proteoglycans and significant water content [117]. Several studies have indicated that ankle cartilage exhibits a higher proteoglycan synthesis rate when subjected to everyday wear, leading to an enhanced repair capacity [105]. In addition, the ankle joint contains fewer catabolic mediators that hinder proteoglycan synthesis [103]. Similar studies have confirmed comparable histopathological differences in the cartilage of the femur and talus [118], providing further evidence of distinct variations in cartilage properties across these joints. However, a discrepancy arises in chondrocytic metabolism because knee cartilage demonstrates a greater anabolic response than hip cartilage [99,108,109,110]. Interestingly, there are differences in protein synthesis between these joints, with knee cartilage exhibiting a more robust repair response than hip cartilage. For instance, COMP protein turnover is greater in knee cartilage than in hip cartilage [108]. These findings suggest that knee cartilage is more biologically resistant than hip cartilage. However, it is important to consider that the knee cartilage is more susceptible to degeneration in the elderly, highlighting the complexity of interpreting OA mechanisms without comprehensive data. Understanding human OA is challenging, and the underlying mechanisms responsible for these differences in degenerative processes remain to be fully elucidated. Although the ankle joint provides better protection against OA than the knees and hips, it is essential to inquire whether these differences in repair response mechanisms are absent or diminished in the early stages of joint involvement. In this regard, the meticulous examination of the diverse variations in articular cartilage thickness could provide key insights into unraveling this mystery. Therefore, a thorough examination of the anatomical diversity of cartilage in weight-bearing joints is crucial for understanding their structural and functional adaptations as well as their unique susceptibilities to OA.

## 8. Cartilotype and Joint Resilience: Thicker Cartilage or Greater Risk? Lessons from the Ankle Paradox

Despite extensive research, the reasons behind the varying vulnerability of different load-bearing joints to OA remain elusive to clinicians, scientists, and public health practitioners. As highlighted previously, the reductionist view that increased mechanical load alone is responsible for OA development must be reconsidered as it oversimplifies the underlying complexities of the disease. Instead, alternative interpretations of the available data are warranted, as significant knowledge gaps remain regarding the synergistic interaction of biomechanical, anatomical, morphological, and metabolic factors in supporting resilient, healthy, and durable joint function. The overall health of the diarthrodial joints, particularly the knee, hip, and ankle, is intricately influenced by this multifaceted interplay of factors [4,5,12]. However, it is important to underscore that the matrix constituents of cartilage in these joints are largely similar, characterized by hydrated proteoglycans and a highly organized collagen structure [111,112,113,114,115]. Additionally, the knee joint is supported by a greater number of surrounding muscles [119], and the articular surfaces of the hip joint are highly congruent [120], indicating that incongruity alone cannot be solely attributed as a determining factor. However, the thickness of the articular cartilage is a commonly overlooked yet crucial factor. The ankle cartilage is notably thin, averaging approximately one millimeter, nearly three times thinner than the knee cartilage [121,122]. The exact effect of differences in cartilage thickness is unknown; nonetheless, these morphometric variations may play a critical role in articular degeneration in weight-bearing joints. It is important to acknowledge that cartilage thickness in larger joints is not directly correlated with joint strength. Although the relatively thin nature of ankle cartilage makes it more vulnerable to collagen damage and mechanical wear [107], thereby increasing the risk of developing OA, it also offers several benefits such as enhanced tissue nutrition, greater resistance to oxidative stress, reduced rates of aging and cell death, and a higher density of chondrocytes. Nutrient supply within the synovial cavity varies according to the size of the diarthrodial joint [34]. Whether chondrocytes in the knee, hip, or ankle cartilage exhibit altered behavior may largely depend on articular architecture.

Analogous to the morphotype concept, Hogervorst et al. [123] introduced the term ‘cartilotype’ to assess the susceptibility or resilience of cartilage in response to mechanical stress and its ability to withstand the development and progression of symptomatic OA dysplasia in the hip joint. This innovative concept can be applied to hyaline cartilage in additional weight-bearing joints in humans. Clinical observations highlight significant disparities in the prevalence and severity of OA between the ankle and femoral joints [42,43]. These thickness-based differences suggest that variations in cartilage zone-dependent properties may play a significant role in determining OA prognosis. Interestingly, while the knee and hip cartilages commonly exhibit pathological or accelerated aging processes, the ankle cartilage demonstrates remarkable robustness by maintaining ongoing matrix turnover throughout life [124]. This remarkable feature highlights the resilient nature of the human talar cartilage, which demonstrates exceptional stability and durability in comparison to the femoral cartilage. As previously discussed, a consistent observation is that the cartilage in human knees and hips typically demonstrates greater thickness in comparison to the cartilage found in the ankles across the majority of anatomical regions. These morphological differences may be related to differences in both metabolic stress and the frequency of OA in these synovial joints. To gain a deeper understanding of the intrinsic disparities that contribute to the development of OA across different weight-bearing joints, it is essential to investigate the substance transport properties within the middle and deep regions of cartilage. The analysis of these transport properties not only sheds light on the intricate physiological mechanisms at play but also provides further insights into the underlying factors that drive the development of OA in specific joints, encompassing diverse morphotypes and unique ‘cartilotypes’ [67,121,122,123,125,126,127].

At this stage of the discussion, a critical question arises: does a larger volume of cartilage contribute to enhanced joint health? Szczodry et al. [28] provided compelling data demonstrating that osteochondral injury is significantly influenced by cartilage thickness. Specifically, under high-impact energies, osteochondral explants with cartilage thinner than 2 mm exhibited a severe fracture rate of 64.7% compared to 27.2% in those with cartilage exceeding that threshold [28]. These findings, discussed in the context of post-traumatic OA, suggest that a thicker cartilage layer may facilitate more effective load distribution and diminish localized stress on both the articular surface and subchondral bone, thereby mitigating micro-damage. However, it remains critical to investigate whether these mechanical advantages translate into a protective effect in idiopathic OA, where degenerative changes occur independently of acute trauma. In parallel, the roles of biochemical and cellular factors—including cell viability, ECM integrity, and overall tissue regenerative capacity—must be integrated into the evaluation. Thus, a comprehensive assessment that includes cell density and other cellular parameters is essential to determine whether increased cartilage thickness truly correlates with enhanced joint health in the long term.

Cartilage thickness and cell density vary across different joints and species, with a consistent trend showing that thinner cartilage is typically accompanied by higher cell density [125,126,127,128]. According to the pioneering study conducted by Stockwell [128], there is an inverse relationship between the cell content (y) measured in cells per mm^3^ and cartilage thickness (x) measured in millimeters in mammalian articular cartilage. This relationship can be mathematically expressed by the concise equation y = 28,000x^−88^. The equation clearly indicates that, as the cartilage thickness increases, the cell content tends to decrease, underscoring the inverse relationship between these two variables. The cartilage found in the weight-bearing regions of the adult human femoral head and condyle exhibits cell densities of approximately 10,000 and 15,000 cells/mm^3^ [129], respectively. In comparison, the cell density in the talar cartilage is around 25,000 cells/mm^3^ [129]. This marked variation in chondrocyte density across different articular sites underscores the importance of local cartilage architecture. Consequently, the morphology and thickness of the articular cartilage have significant implications for the organic state of the diarthrodial joint, directly influencing the chondrocyte nourishment and waste removal processes. Synovial fluid plays a crucial role in providing essential nutrients to the cartilage and eliminating the metabolic waste products necessary for maintaining cell viability [37]. However, it is essential to acknowledge that nutrient supply and byproduct elimination mechanisms in the thicker cartilage of knees and hips may not be adequately adapted to cope with the challenges posed by modern sedentary lifestyles. Although the underlying mechanisms remain unclear, it is evident that functional degeneration occurs more rapidly in the knee and hip joints than that in the ankle joint. This emphasizes the vital importance of incorporating cartilage thickness into the investigation of musculoskeletal disorders, such as OA; overlooking this critical factor not only oversimplifies the intricate pathoetiology but also compromises the development of effective treatment strategies.

## 9. Limiting Depth: Role of Joint Motion in the Nutrition of Articular Cartilage

Generally speaking, the limiting depth represents a physical-chemical impediment that defines the permeability of hyaline cartilage under passive diffusion conditions [31,32,36], and has significant biological implications for OA pathogenesis. Despite being an old territory in cartilage research, the importance of limiting depth deserves further investigation. Limiting depth refers to the point at which nutrient concentrations drop steeply with distance from the cartilage surface, resulting in slower metabolism of chondrocytes in the middle and deep layers. Articular cartilage, a tissue without its own blood supply, relies on synovial liquid that surrounds the joint cavity for nutrition [34,37]. However, the amount of nutrients that can reach the cartilage through the synovial fluid is limited and inversely proportional to the cell density in a given area of the tissue. Logically, the presence of thicker cartilage with a higher cell content corresponds to a lower nutrient supply to each individual cell. In synovial joints, this delicate balance between cartilage thickness and cell content is critical for preserving the overall health and functionality of articular cartilage, which requires continuous nutrition to maintain its unique biological and mechanical properties [31,32,37,128]. Chondrocytes rely on synovial liquid as their primary source of nutrients, and the fluid also helps sustain the viability of chondrocytes located in the deep zone. These chondrocytes primarily depend on the diffusion of nutrients from synovial fluid to meet their nutritional needs. Over time, the integrity of the cartilage may be compromised, as deep zone chondrocytes, which depend on diffusion-based synovial fluid nutrition [37], may not be able to survive.

As discussed above, the concept of limiting depth is pivotal in understanding nutrient transport within joint cartilage, a tissue that lacks its own blood supply and relies entirely on diffusion from synovial fluid to sustain chondrocytes embedded within its dense, gel-like matrix. This limiting depth refers to the maximum distance nutrients such as glucose and oxygen can diffuse into the cartilage before they are consumed, and is mathematically expressed by the equation:

[d = √(2PC_o_/Q)] (1)

where d is the limiting depth, P is the diffusion coefficient of glucose, C_o_ is the concentration of glucose in synovial fluid, and Q is the rate of glucose utilization by chondrocytes [31,32]. This equation shows that the diffusion of glucose into cartilage is directly influenced by its diffusion capacity (P) and availability in the synovial fluid (C_o_), but is constrained by the metabolic demand of the chondrocytes (Q). Therefore, any factor that alters these parameters can significantly influence the depth to which glucose reaches chondrocytes, especially in the deeper regions of the cartilage. As glucose demand increases or availability decreases, the depth to which these molecules can diffuse becomes limited, potentially depriving cells in deeper zones of sufficient resources for survival and matrix maintenance. The implications of this for cartilage health are significant, particularly in thick weight-bearing joints, such as the knee and hip, where diffusion distances are greater. In these joints, if the limiting depth is insufficient relative to cartilage thickness, deep chondrocytes may experience nutrient deprivation, leading to reduced cellular activity, impaired ECM synthesis, and eventual cartilage degradation.

**Figure 3:**
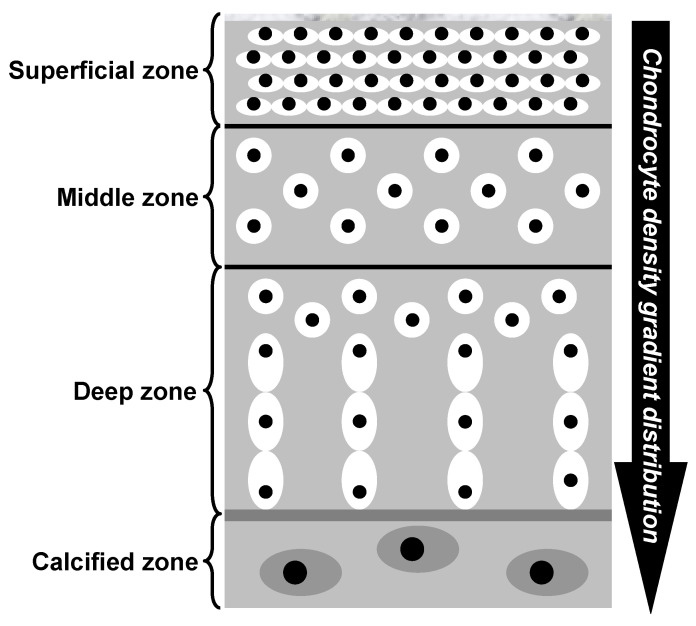
Histomorphological characteristics of hyaline cartilage in adult mature diarthrodial joints (shown diagrammatically). Cartilage is a specialized connective tissue composed of a small number of chondrocytes embedded in an abundant ECM dominated by type II collagen and large aggregating proteoglycans. Articular cartilage demonstrates a highly heterogeneous, multi-layered arrangement of chondrocytes and ECM, exhibiting depth-dependent anisotropic behavior—reflecting its transverse isotropy—in both solid and fluid displacement. This complex structural organization underscores the functional specialization of the tissue. Chondrocytes—comprising fewer than 5% of the tissue volume—are involved in the formation of distinct zones in the cartilage, including superficial, middle, deep, and calcified zones. Depicted as a thick gray line, the tidemark represents a critical boundary marking the transition to calcified cartilage, which is essential for preserving the structural integrity and stability of cartilage. Although not shown in this figure, the lamina splendens of cartilage features an acellular upper layer—measuring approximately 3 to 8 µm in thickness and primarily composed of collagen fibrils—along with essential lubricating agents such as lubricin, surface-active phospholipids, and hyaluronic acid, which together reduce friction and support joint function. The key difference lies in the number of cells that each zone of the joint cartilage can accommodate. Along with proteoglycans and collagen fibers, chondrocytes on the nutrient-rich surface of cartilage—where glucose and oxygen are readily available—act as a protective barrier for cells located deeper within the tissue. The limiting depth defines the maximum depth to which these nutrients can penetrate the cartilage matrix from the synovial fluid, ensuring that cells deep within the tissue remain viable. If the limiting depth is less than the full cartilage thickness, deeper chondrocytes may experience nutrient deprivation, leading to metabolic imbalance and compromised maintenance. Adapted from [133].

Hyaline cartilage is a remarkably intriguing tissue, characterized by being avascular, aneural, alymphatic, and typically hypocellular, with a sparse population of cells embedded within a highly negatively charged ECM [111,114,121]. Cartilage exhibits a depth-dependent, non-homogeneous zonal organization in which planes parallel to the articular surface are relatively uniform, whereas the matrix composition and collagen fiber orientation vary perpendicular to the surface. This stratification underlies functional specialization— the superficial zone provides shear resistance, whereas the deeper zones confer compressive load-bearing capacity. As shown in Figure 3 and detailed in Table 1, cartilage demonstrates distinct gradients in cell density and concentration, with a higher abundance of chondrocytes observed in the superficial zone compared to the middle and deep regions [130]. The figure illustrates the histomorphology of the human cartilage, where the superficial zone has a dense concentration of flattened chondrocyte cells arranged parallel to the articular surface. In contrast, the middle zone exhibits less organized and round chondrocytes with a lower concentration. The deep zone contains larger chondrocytes organized in vertical columns perpendicular to the surface, with the lowest concentration of cells. Consequently, the superficial zone of the articular cartilage is likely to utilize a substantial portion of the available nutritional resources. Mitochondria, essential for glucose metabolism [131,132], may face challenges in meeting the energy demands of deep cartilage as its thickness increases. In simple terms, the implication of the above explanation is that the superficial layer of cartilage may have a relatively greater reservoir of nutrients within its ECM, making it less reliant on joint motion and synovial fluid for nourishment. In contrast, deeper layers of cartilage may have a higher dependence on joint movement and synovial fluid for nutrient supply. This highlights the differential importance of synovial fluid diffusion in providing nourishment to the deep zone, as it plays a crucial role in delivering essential nutrients to maintain cartilage health and functionality. These differences in nutrient supply and dependence on synovial fluid diffusion may have significant implications on the susceptibility of human joints to OA, as compromised nutrient availability in the deep zone may contribute to the development and progression of the disease [37]. This emphasizes the need to understand and address these differences to preserve the viability of cartilage cells and their ECM.

However, why does a greater cartilage thickness increase the vulnerability of the deepest cartilage cells? In essence, articular cartilage is composed of phenotypically distinct zones, including superficial, middle, deep, and calcified zones [80,114,115,121,130,133]. Each zone is characterized by unique arrangements of chondrocyte cells, as illustrated in Figure 3. This zonal organization plays a crucial role in determining the functional properties and structural integrity of the cartilage tissue. In medium- and large-sized mammals, the cell densities of the superficial and deep layers of the cartilage exhibit substantial contrast [127,128,130,134,135]. The superficial layer demonstrates a considerably higher density of 1.5 × 10^8^ cells/cm^3^, whereas the deep layer displays a lower density of 5.0 × 10^7^ cells/cm^3^. However, this difference is not by chance. In the superficial layer, chondrocytes and collagen are densely packed [136], forming a layer commonly called the *armor plate* (see Table 1, for details). Both the cartilage microarchitecture and the nanostructure of the three-dimensional chondrocyte matrix were conceived under the same chondroprotective criteria. From the lamina splendens to the chondrons, the matrix components are intricately organized to function as both a “filter” against biochemical threats and a “shield” against the compressive, tensile, and shear forces that constantly impact the articular cartilage, establishing a cohesive protective barrier that extends from the superficial zone to the chondrocyte cells [134,137,138,139]. Quantitative analysis reveals that the physiological domain—defined as the average matrix volume maintained by individual chondrocytes—increases markedly from approximately 45,000 μm^3^ in the superficial zone to nearly 130,000 μm^3^ in the deep zone [134]. Consequently, deeper cartilage layers present a significant challenge, with few cells, restricted nutrient availability, and a substantial ECM that needs to be maintained, leading to evolutionary failure [69]. This inherent limitation in the deep layers of cartilage makes diarthrodial joints with thick cartilage more susceptible to OA due to metabolic imbalance, especially after reproductive age. Nevertheless, the degree of susceptibility is closely related to the magnitude and frequency of joint movement involved.

Pioneering studies have demonstrated that the maintenance of adult human cartilage depends on two fundamental mechanisms: passive diffusion of solutes from synovial fluid and an active “pumping effect” facilitated by cyclic mechanical loading [31,33]. These two mechanisms are achieved through vigorous agitation of the synovial fluid in the joint cavity and the application of cyclical pressure, respectively. The first mechanism, essential for transporting basic nutrients such as glucose and oxygen, can be achieved through joint movement without the need for weight-bearing actions like walking, jumping, or running. The second mechanism, facilitated by mechanical loading, assumes greater importance in the transport of high-molecular-weight molecules, including growth factors, enzymes, and hormones. Maroudas et al. [31] examined the effects of joint activity on nutrient diffusion using animal cartilage, which closely approximated the thickness of the human cartilage. The experiment involved injecting dye into one mobile and one immobile joint in the same animal and observing the rate of dye diffusion into the cartilage after immersion in a stirred dye solution for forty-five minutes. The results revealed that the diffusion rate was significantly lower in the immobile joint, indicating that both cartilage and liquid offer resistance to material transfer, which affects the overall diffusion rate. Although efficient stirring of the fluid can maintain a uniform concentration of nutrients, a thin stagnant film remains at the interface [31,33]. These pioneering studies provide unequivocal evidence of the crucial role of joint movement in stimulating synovial fluid, thus emphasizing its indispensable function in delivering essential nourishment to articular cartilage.

Upon comprehensive examination of these fundamental concepts, it becomes evident that the nourishment of articular cartilage is a complex and intricate process characterized by a slow and non-uniform distribution of nutrients. In addition, Levick [34] reported that nutrients are supplied to synovial fluid by synovial capillaries. However, diffusion from these capillaries through the thin synovial fluid film to the center of the cartilage surface is not sufficient to nourish the cartilage properly. In immobilized large joints, such as the knee and hip, the lack of joint movement-induced stirring of synovial fluid provides a plausible explanation for the premature degradation of cartilage. Undoubtedly, this highlights the importance of regular joint motion in promoting the health and longevity of cartilage tissue. Joint motion promotes cartilage nutrition by introducing convective transport as it stirs the synovial fluid layer. The limitation of diffusion in cartilage thickness is evident as the normal rate of glycolysis falls to zero at 2.2–3.2 mm [34]. This almost doubles to 4–6 mm when accounting for the non-uniform distribution of chondrocytes. The thickness of hyaline cartilage in large human joints is typically 2–4 mm [121,122], leaving a narrow safety margin for the nutrition of deep chondrocytes, even in a well-mobilized joint. Hence, it is highly probable that joint motion plays a significant role in facilitating intra-articular transport, thereby playing a crucial role in ensuring adequate cartilage nutrition. These findings strongly suggest that thicker cartilage is more challenging to supply with nutrients than thin cartilage, potentially explaining why the ankle joint is more resistant to degenerative OA than the hip or knee joint, despite experiencing higher load-bearing pressures per square millimeter. In light of this evidence, it can be tentatively concluded that the greater requirement for joint motion and increased vulnerability to nutritional stress experienced by thicker cartilage may play a significant role in the elevated prevalence of OA in weight-bearing knee and hip joints. To strengthen this preliminary conclusion, I will conduct further investigations focusing on the paradox observed in the human ankle joint, as discussed earlier, to provide valuable insights and enhances our understanding of the underlying factors involved.

## 10. Examining the Role of Limiting Depth and Cartilage Thickness in Osteoarthritis Development

In light of the preceding information, the relationship between limiting depth and cartilage thickness plays a critical role in elucidating the mechanisms underlying OA development and progression. Urban et al. [140] have made significant contributions to our understanding of intervertebral disc degeneration by elucidating the complex relationship between nutrient availability, cellular viability, and the underlying mechanisms driving the pathogenesis of degeneration caused by the loss of nutrient supply. Similarly, pioneering research conducted by Maroudas et al. [31,33] has played a crucial role in advancing our knowledge by providing key insights into the nutritional environment of chondrocytes in the hyaline cartilage of weight-bearing joints. By examining the nutrient transport mechanisms, these studies have deepened our comprehension of the role of nutrient availability in cartilage degeneration. Together, these combined efforts have significantly enhanced current understanding of the pathogenesis of hyaline cartilage degradation, establishing a solid foundation for future research and development of innovative therapeutic interventions [141]. As previously mentioned, weight-bearing synovial joints, such as the knee and hip, exhibit increased cartilage thickness that serves a critical mechanical purpose in providing essential support through the presence of a larger ECM [121,122,125]. Shepherd et al. [122] found that thicker cartilage is associated with a lower compressive modulus, which is attributed to its increased ability to deform under pressure. This phenomenon leads to an expanded contact area between the incongruent joint surfaces, which effectively reduces stress levels to an acceptable range. However, this thicker cartilage configuration also imposes limitations on the depth of the tissue that can be adequately nourished by synovial fluid. The presence of high cell density in the superficial layer hinders the diffusion of nutrients to deeper regions, thereby affecting proper nourishment. In contrast, the ankle joint features a relatively thin articular cartilage [122], facilitating the efficient diffusion of nutrients and oxygen. As a result, chondrocytes in the ankle joint can maintain tissue health and integrity with less effort compared to thicker cartilage. Unlike the knee and hip joints, thinner cartilage in the ankle joint allows for improved nourishment by synovial fluid, supports a larger population of chondrocytes, and contributes to tissue homeostasis and structural integrity. Understanding these variations in cartilage thickness and their implications for joint health is critical for the prevention and treatment of OA, particularly in femoral joints.

**Figure 4:**
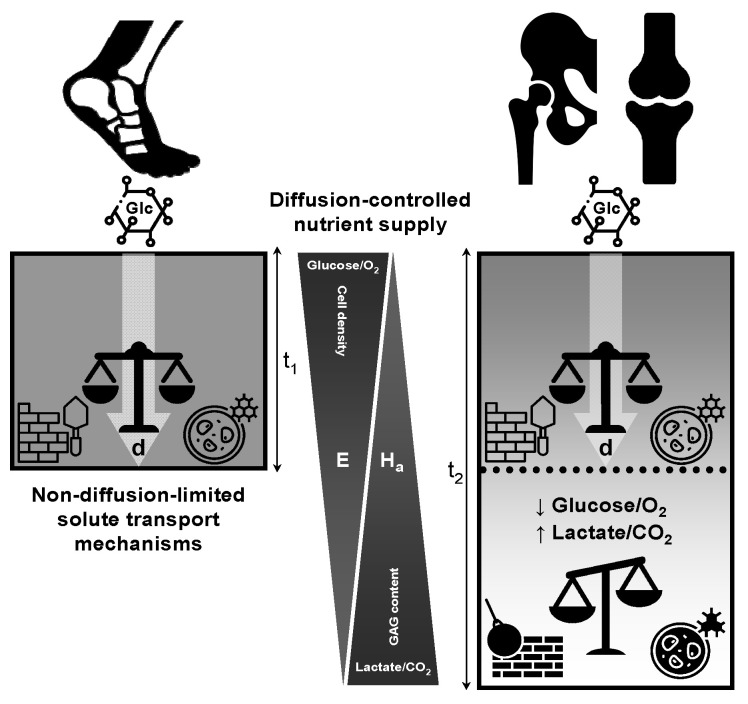
Two hypothetical scenarios for bioactive solute transport in stationary (non-loaded) hyaline cartilage. Both cases assumed no joint motion and thus no convective mixing (i.e., purely diffusive transport). The left panel shows a thin cartilage layer (t_1_ ~ 1.3 mm) in which homogeneous glucose diffusion sustains chondrocyte metabolism despite enhanced hypoxic stress, reflecting inherent adaptation to a physiological hypoxic (physioxic) environment. The right panel illustrates a thicker cartilage layer (t_2_ ~ 2.5 mm), in which nutrient supply becomes insufficient beyond the limiting depth (d ~ 1.5 mm from the surface), leading to disrupted anabolic pathways and compromised chondrocyte viability in deep zones. These findings underscore the critical importance of nutrient supply in sustaining chondrocyte survival in the deeper layers of the joint cartilage, with significant implications for overall tissue health. The thickness values were based on the average ankle and knee measurements in a 70 kg individual. Data were obtained from the following references [122,137,142]. Abbreviations: Glc, glucose; t, cartilage thickness; d, limiting depth; E, Young’s modulus; H_a_, aggregate modulus.

Based on the evidence presented in this study, the remarkable resistance of the ankle joint to degenerative OA may be attributed to several factors, including a high index of joint congruence, metabolic factors, as well as the presence of comparatively thin cartilage. Additionally, the ankle joint may benefit from the higher regenerative capacity of cartilage repair associated with the increased cellularity observed in thinner cartilages [127]. Importantly, the virtually nonexistent limiting depth within the ankle joint suggests its lower vulnerability to OA development resulting from nutritional stress in deeper layers of cartilage. This contrasts with the weight-bearing knee and hip joints, where a notably significant limiting depth may occur, particularly in cases of reduced joint activity. A sedentary lifestyle that restricts joint movement and disrupts the delivery of synovial fluid can have detrimental effects on the cartilage. This limitation hampers nutrient flow and waste removal, contributing to cartilage degeneration and eventual development of OA. Given the growing prevalence of sedentary lifestyles, OA should not be viewed primarily as a result of biomechanical stress but rather as a condition driven by nutritional deficiencies associated with the limiting depth. In this context, prolonged periods of restricted joint movement can compromise nutrient delivery to the cartilage cells, particularly in the knees and hips of sedentary individuals, by impairing synovial fluid circulation and reducing metabolic exchange [37]. The diffusion time of nutrients is proportional to the square of the distance they must travel, and it is further slowed down by consumption along the diffusion route, which makes it harder to supply nutrients to thicker cartilages. This suggests that OA is likely to be less prevalent in the ankle joint compared to the knee and hip joints, primarily attributable to disparities in cartilage transport properties. According to studies conducted by Maroudas et al. [31,33], it has been suggested that the limiting depth in the ankle joint exceeds the thickness of the cartilage, thereby preventing uneven degradation that could lead to the loss of components within the deep cartilage. This feature is a hallmark structural phenotype of established OA in the knee and hip joints. Despite being subjected to higher load pressures per mm^2^, the ankle joint demonstrates remarkable resistance to degenerative OA, primarily attributed in this study to the efficiency and thinness of its cartilage. This suggests that cartilage in the ankle joint is typically characterized by robust nourishment and efficient waste removal mechanisms, as illustrated in Figure 4. These inherent characteristics are postulated to enhance the resilience of the ankle cartilage against nutritional stress and diminish the accumulation of waste in deeper layers, even during periods of rest. Furthermore, it is proposed that the ankle joints demonstrate a reduced reliance on joint motion compared to the femoral joints, indicating a potentially distinct mechanism for sustaining cartilage health and optimal functionality. This intriguing finding provides a compelling explanation for the paradox observed in the human ankle, where it remains resilient to OA despite the substantial load pressures it experiences. Therefore, while mechanical stress is widely recognized as a destabilizing factor, the thickness of the articular cartilage may also play a significant role in the development of OA, especially in weight-bearing joints, such as the knee and hip.

## 11. Bridging the OA Puzzle: Could Stagnant Synovial Films Be the Missing Key to Cartilage Degeneration?

Despite being recognized as an avascular tissue for over two centuries [143], the underlying reason why sustained joint movement is critical for the long-term survival of thick human cartilage remains largely unexplored. In this context, an important factor that has been consistently overlooked in previous studies is the impact of stagnant liquid films on the surface of cartilage [31,33]. These stagnant films or unstirred layers, which have received limited attention thus far, have the potential to significantly influence the development and progression of OA in the knee and hip joints. As previously mentioned, cartilage comprises chondrocytes responsible for preserving the ECM, whereas synovial fluid—beyond its lubricating role—delivers essential nutrients and facilitates waste removal to support tissue function, metabolic stability, and structural integrity [37]. In turn, chondrocyte nourishment and survival require that the supply of sufficient exogenous substrates provided by the synovial fluid meets their metabolic demands, thereby supporting the biosynthetic pathways essential for cellular homeostasis and matrix turnover. The profile of macromolecules synthesized and secreted by chondrocytes—primarily type II procollagen (the precursor to type II collagen), aggrecan (a proteoglycan rich in chondroitin- and keratan-sulfate glycosaminoglycans), hyaluronan, and multiadhesive glycoproteins (e.g., fibronectin, cartilage oligomeric matrix protein)—underscores their dependency on substrates such as glucose, amino acids, and inorganic sulfate [144,145]. However, disruptions in the normal flow of synovial fluid can lead to an ‘unstirred layer’ effect that impedes molecular transport, thereby limiting the efficient exchange of nutrients and waste products within the joint cartilage. This disruption can lead to the accumulation of harmful substances and result in cartilage damage over time.

**Figure 5:**
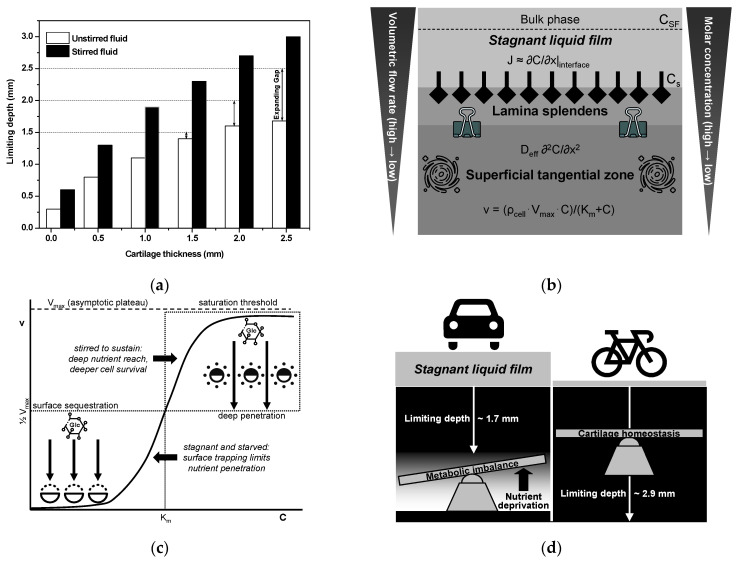
(**a**) Dependence of glucose supply limiting depth on cartilage thickness in the femoral condyle under stirred and non-stirred synovial fluid conditions. The bars represent the critical diffusion depth, beyond which chondrocyte metabolism becomes nutrient-limited, computed for increasing cartilage thickness. Under non-stirred conditions (white bars), the limiting depth falls below the true cartilage thickness beginning at ~1.5 mm (dashed line), marking the threshold for glucose-deprived zones. In contrast, stirred conditions (black bars) maintain a supply depth that always exceeds the tissue thickness. The widening “transport gap” (i.e., the vertical distance between the stirred and non-stirred bars) emphasizes the increasingly critical role of convective mixing as the cartilage thickness increases. Parameters: cell density ρ ~ 10^7^ cells/cm^3^; glucose diffusion coefficient D = 2.7 × 10^−6^ cm^2^/s; glycolytic rate (assuming a standard cell density) Q = 10^−7^–10^−6^ mol/cm^3^·h. Data from [146] (**b**) Schematic cross-section of the articular surface, showing three successive layers (from the joint space inward): a stagnant liquid film (unstirred synovial layer), which acts as a quiescent fluid-film bearing surface, impeding replenishment of fresh, plasma-derived nutrients from the bulk synovial fluid and thereby reducing the passive diffusion rates of low-molecular-weight solutes despite their high intrinsic diffusivity; the lamina splendens, an acellular, amorphous collagenous coat that provides an ultra-low friction interface and a low-permeability barrier; and the STZ, characterized by type II/IX collagen fibrils aligned tangentially to the surface, interstitial water, and proteoglycans. STZ mediates tensile load transfer, shock absorption, and boundary lubrication (via lubricin, surface-active phospholipids, and hyaluronic acid), thereby protecting the subchondral bone—its disruption markedly decreasing cartilage load-bearing capacity and increasing subchondral strain. (**c**) Schematic model illustrating the interplay between GLUT-mediated transport kinetics and depth-dependent glucose penetration in avascular hyaline cartilage. Michaelis–Menten curves plot transport velocity (v) as a function of extracellular glucose concentration (C) for key chondrocyte glucose transporter isoforms (e.g., GLUT1 and GLUT3), each defined by characteristic K_m_ and V_max_ values. At low glucose levels or sub-saturating concentrations (C ≪ K_m_), uptake increases nearly linearly with C, enabling superficial zone cells—expressing primarily high-capacity GLUT1 and high-affinity GLUT3—to sequester most incoming glucose and generate a steep depletion gradient, thereby limiting penetration to upper zones. As C approaches K_m_, uptake begins to saturate, and for C ≫ K_m_, transporter sites are fully occupied, and v asymptotically approaches V_max_, marking the saturation regime where further increases in C do not enhance uptake. This nonlinear uptake behavior (or saturation effect) reduces glucose consumption in the superficial layer, allowing the excess substrate to diffuse deeper into the tissue. The accompanying depth profiles illustrate this transition: under low synovial glucose, sub-saturating uptake by superficial transporters remains unsaturated in the superficial layer, restricting glucose to shallow regions; under high glucose, saturation of surface transporters allows enhanced diffusion into the middle and deep zones, thereby increasing the effective nutrient penetration depth. This model integrates the finite transport capacity of GLUT isoforms with ECM diffusion constraints to explain the depth-dependent availability of glucose in the avascular cartilage. Abbreviations: v, transport velocity; C, extracellular glucose concentration; K_m_, half-saturation constant; V_max_, maximum transport rate. (**d**) Representative diagram illustrating the effect of joint motion on the limiting depth of the articular cartilage by agitation of synovial fluid. The left panel represents the formation of an unstirred layer on the cartilage surface during periods of rest, indicating film-diffusion-controlled conditions. In contrast, the right panel illustrates synovial fluid that is actively agitated during joint movement, creating a dynamic environment that promotes efficient nutrient exchange, waste removal, and optimal maintenance of cartilage health. The studies in refs [31,32,147] provided data on cartilage thickness and limiting distance, as shown in the figure, which illustrates the nourishment received from the articular surface source in the human femoral head.

As discussed earlier, the limiting depth is defined as the distance from the articular surface at which the concentration of glucose (and other small solutes) falls below the threshold required to sustain normal chondrocyte metabolism, or equivalently, where cellular uptake demands exceed the diffusive supply. Figure 5a presents a bar chart of the limiting depth (in millimeters) plotted against the total cartilage thickness under both unstirred and stirred synovial fluid conditions [146]. Under unstirred conditions, the viable zone falls short of full thickness by 0.20 mm (13.3%) at 1.5 mm total thickness, by 0.40 mm (20.0%) at 2.0 mm, and by 0.80 mm (32.0%) at 2.5 mm. This imbalance between cellular uptake and diffusive supply intensifies with increasing depth, creating a metabolically inactive “dead zone”. In contrast, stirring the stagnant film maintains full-depth viability, effectively preserving nutrient access throughout the tissue [146]. Consequently, in hyaline cartilage that exceeds approximately 1.5 mm in thickness, the pathophysiological impact of limiting depth can be significantly exacerbated by the sedentary behaviors prevalent in contemporary societies. In contrast, thinner cartilage reduces the diffusion distance, rendering peripheral joints, such as the ankle, less susceptible to stagnant film-related nutrient deficiencies. Except where the cartilage is extremely thin (Figure 5a), the concentration gradient becomes sufficiently abrupt, and cellular metabolism is progressively compromised as the cartilage thickness increases. Beyond the critical 1.5 mm threshold, these data reveal that, in sedentary individuals whose synovial fluid remains largely unstirred, even modest increases in cartilage thickness give rise to disproportionately large hypometabolic zones. Although available data are currently limited to a maximum thickness of 2.5 mm [146], projecting these quantitative trends to cartilage that often exceeds 3 mm in the knee and hip predicts even greater absolute and relative deficits under unstirred conditions, rendering these load-bearing joints particularly vulnerable to nutrition-limited degeneration in low-mobility populations. This analysis underscores the importance of regular joint motion—and the associated thinning of the unstirred synovial layer—in preserving nutrient flux and deep zone chondrocyte viability.

According to classical mass-transfer and ion-exchange kinetics theories for heterogeneous membranes [142,148,149], unstirred-layer thicknesses typically range from 10^−3^ to 10^−2^ cm under stirred conditions yet can increase substantially when convective mixing ceases. Understanding this range (see Table 2 for details) is critical, as fluctuations in stagnant film thickness can dramatically affect the efficiency of nutrient and waste exchange, ultimately influencing cartilage health and functionality. In diarthrodial joints, where the articular cartilage is entirely avascular, the quiescent “stagnant” synovial-fluid film lining the surface represents the primary barrier to nutrient delivery. As shown in Figure 5b, classical film theory predicts the diffusive molar flux J across this unstirred layer, given by Fick’s first law [150]:

[J = D/δ (C_SF_ − C_s_)] (2)

where D is the molecular diffusion coefficient, δ is the film thickness, and C_SF_ and C_s_ are the solute concentrations in the bulk synovial fluid immediately at the cartilage interface, respectively. In the absence of convective flow (or renewal), the concentration gradient (ΔC) decays until no net transport remains, severely limiting the supply of low-molecular-weight nutrients to chondrocytes. The thickness δ of this stagnant film, and thus, the rate of nutrient flux, is dynamically governed by three key factors. First, flow regime and stirring intensity, whereby joint motion or synovial mixing thins the film, but rest allows δ to grow. Second, fluid viscosity, because higher viscosity mixing-induced suppresses turbulence and thickening of the boundary layer; indeed, hydrodynamic theory shows δ→0 only in the hypothetical limit of vanishing viscosity under sustained agitation. Third, diffusion coefficient itself influences the effective barrier; according to the laminar boundary-layer theory (δ∝D^1/3^), the faster-diffusing molecules paradoxically experience a relatively thicker unstirred region. Together, joint kinematics, synovial-fluid rheology, and solute molecular size govern the effective permeability of the cartilage–synovial interface and the efficiency with which essential metabolites diffuse across the unstirred film to maintain cartilage health. Conversely, any reduction in film renewal (e.g., joint immobilization) or increase in viscosity (e.g., during inflammatory synovitis) can critically impair nutrient transport to chondrocytes, accelerate ECM degradation, and osteoarthritic progression [37].

Articular cartilage, long thought to possess an almost perfectly smooth surface, in fact exhibits reproducible microscale depressions—tertiary hollows of approximately 20–30 µm diameter and 0.5–2 µm depth—that deepen with maturation (from ~0.6 µm in immature tissue to >1 µm in adults) and function far beyond mere surface imperfections [32]. Rather, it is hypothesized that these hollows act as micro-reservoirs that trap synovial fluid under compressive and sliding loads, sustaining a thin lubricating film that minimizes asperity contact and promotes a localized hydrodynamic lift. Their size and spacing also facilitate the “micro-pumping” of fluid into the contact interface, enhancing both fluid film and boundary lubrication. Concurrently, by disrupting the otherwise laminar synovial boundary layer, tertiary hollows may induce microturbulence that accelerates the diffusion of oxygen, glucose, and metabolic waste across the superficial 100–200 µm critical for chondrocyte viability [146]. Moreover, cyclical loading–unloading cycles generate microscale pressure gradients that further drive fresh fluid into the tissue, whereas a perfectly smooth or irregularly protruding surface fosters laminar flow, stagnation, and nutrient deprivation. Importantly, the maintenance and sculpting of these microfeatures depend on dynamic joint movements. Although excessive hollow depths may signal early degeneration, a moderate activity-dependent contour appears integral to cartilage homeostasis. Indeed, it is plausible that sedentary behavior, by eliminating the cyclical shear and compression needed to sculpt and maintain these microfeatures, could flatten hollows or result in the persistence of surface irregularities, compromising lubrication and impeding nutrient exchange, thus accelerating chondrocyte malnutrition and wear. Elucidating the mechanisms underlying tertiary hollow remodeling through advanced imaging, tribological modeling, and mechanobiological studies is therefore essential for developing strategies that preserve cartilage microarchitecture and translating these insights into preventive and therapeutic approaches against OA.

The lamina splendens, the uppermost few microns of the superficial tangential zone, comprises a highly stiff, ultra-low-permeability collagenous barrier that regulates interstitial fluid pressurization and solute diffusion into the cartilage (see Figure 5b; Table 2). This specialized layer consists of a nearly pure, tightly woven network of randomly oriented collagen fibrils saturated with interstitial water yet depleted of proteoglycans, rendering it substantially stiffer and less permeable than the deeper, proteoglycan-rich zones. Torzilli et al. [36] suggested that the lamina splendens serves not only as a superficial boundary of the articular surface but also as a critical structural element that maintains the cohesion of the underlying matrix by mechanically supporting and constraining the highly hydrated, sulfated proteoglycans within the superficial cartilage zone. Under compressive loading, the superficial tangential zone (STZ) exhibits greater expansion than the underlying layers; however, the potential collapse of this water-rich, proteoglycan-poor mesh may dramatically disrupt the transport of solutes across the articular surface, thereby impairing nutrient exchange and waste removal essential for chondrocyte function. The removal or fibrillation of this layer, whether by early osteoarthritic degeneration or iatrogenic abrasion, exposes water-swollen proteoglycan macromolecules to the joint space, where they expand and form an entangled network that further hinders solute diffusion [36]. This dual disruption of mechanical integrity and solute transport—for both nutritional (e.g., oxygen and glucose) and therapeutic (e.g., intra-cartilage drugs) molecules—can reduce mid-zone solute concentrations, starve chondrocytes, and trigger a vicious cycle of matrix degradation. These findings underscore that the lamina splendens functions not merely as a wear-resistant “skin” but as a critical biophysical regulator of both mechanical stability and molecular transport, highlighting the imperative to preserve or restore its architecture to support nutrient supply, effective drug delivery, and long-term cartilage integrity.

In the avascular environment of articular cartilage, chondrocytes meet their high glycolytic demand—required for ATP production and matrix synthesis—through the coordinated expression of multiple facilitative glucose transporters (GLUTs) [144]. These include high-capacity GLUT1 for basal uptake, high-affinity GLUT3 for low-concentration scavenging, and auxiliary transporters GLUT6 and GLUT9 to fine-tune delivery under varying conditions. Glucose diffuses from the synovial fluid into the ECM, characterized by an effective diffusivity (D_eff_), and is consumed by superficial chondrocytes following the Michaelis–Menten kinetics [151]. When pericellular glucose is below K_m_, uptake rises nearly linearly with concentration, allowing surface cells to capture most available substrates and establish a steep depletion gradient that limits penetration depth (Figure 5c). As synovial glucose approaches or exceeds K_m_, transporter sites become saturated, and uptake plateaus at V_max_, permitting excess glucose to diffuse deeper into the tissue. This non-linear, isoform-specific saturation behavior generates a limiting depth that is superficial under low-glucose conditions and extends under high-glucose conditions. This limiting depth is further modulated by zonal variations in matrix porosity, chondrocyte density, and the presence of alternative metabolic pathways. Thus, the concept of a “limiting depth” emerges from the interplay between diffusive transport and transporter saturation: low glucose yields a shallow penetration front dictated by high-capacity uptake at the surface, whereas higher glucose concentrations push the penetration front deeper once surface transporters are saturated [31,32,33,34,35]. Conceptualizing nutrient zonation as the convergence of diffusion constraints and kinetic ceilings of GLUT-mediated uptake provides a qualitative mechanistic framework for understanding depth-dependent chondrocyte viability and matrix homeostasis. This framework highlights how both synovial fluid composition and transporter kinetics collaborate to shape the metabolic microenvironment of chondrocytes across cartilage depth.

Figure 5d schematically illustrates how physical exercise enhance nutrient delivery from synovial fluid, minimize the formation of the unstirred boundary layer, and maintain thick cartilage health, thus addressing the challenges associated with tissue-depth limitations. This figure provides a graphical representation of how joint motion influences the limiting depth of cartilage, directly affecting the characteristics of the unstirred layer. Joint movement, facilitated by physical activity/exercise, plays a crucial role in promoting the flow and mixing of synovial fluid, which is essential for preventing the formation of stagnant liquid films and maintaining optimal cartilage health. These unstirred layers act as self-limiting barriers, hindering nutrient transport and proper waste removal from the cartilage, as suggested by Maroudas et al. [31,33]. However, the thickness of these stagnant films can be reduced under the optimal conditions of synovial fluid agitation during joint movement. This understanding, in line with the findings of Maroudas, emphasizes the importance of joint motion in both the prevention and treatment of OA. In this context, the stagnant liquid film creates an isolating barrier covering the articular cartilage surface, effectively separating it from synovial fluid [31,32,33,34,35,128,152,153]. This poses significant challenges for the nutrition and waste removal of cartilage, as the unstirred layer in static joints introduces considerable resistance to diffusion, impairing the effective transport of nutrients and metabolic byproducts into and out of the cartilage, as illustrated in the static transport regime (Figure 5d, left). However, under enhanced convective mixing achieved by optimal synovial fluid agitation, the diffusion-resistant boundary layer decreases in thickness. Film diffusion control is favored in situations characterized by inefficient agitation, which leads to the formation of thick films. Nevertheless, during joint movement, the synovial fluid is stirred, preventing the limiting depth from becoming significant, as shown in the dynamic transport regime (Figure 5d, right). In this scenario, the limiting depth is essentially equivalent to the average actual thickness of the human femoral cartilage because the presence of a well-stirred fluid can disturb the stagnant liquid film [31]. As a result, film diffusion control and generation of unstirred layers are likely negligible factors in this specific case.

Synovial fluid plays a critical role in lubricating and nourishing articular cartilage [37], and agitation has been shown to increase the depth at which essential substances can penetrate cartilage. In the synovial joints, the transport of essential nutrients primarily occurs through diffusion, a physical process that enables the movement of molecules without requiring an external energy supply [150]. This passive process is driven by a concentration gradient, where molecules move from a region of low concentration to high concentration until they reach a dynamic equilibrium, as per the second law of thermodynamics. The speed at which cartilage is nourished is proportional to the movement of synovial fluid, which prevents the formation of stagnant fluid on the cartilage surface, making the nutritional process possible [31,33]. However, limited joint movement can result in cartilage malnutrition by restricting the availability of essential nutrients. This results in a nutrient concentration gradient within the stagnant fluid film, with the concentration falling towards the cartilage interior. Chondrocyte metabolism exacerbates this gradient because the utilization of substances is primarily concentrated near the articular surface, resulting in reduced availability for diffusion into the deeper layers of the cartilage. Consequently, the absorption of nutrients by chondrocytes is hindered unless there is sufficient movement-induced synovial fluid agitation, which prevents the formation of stagnant layers on the cartilage surface [31,33,34,35,128,152]. Understanding the influence of joint movement and synovial fluid flow on cartilage health and function is crucial for developing strategies to promote joint health.

Glucose is the primary energy source for chondrocytes [131,144,145], particularly in the hypoxic deep zone of avascular cartilage, where anaerobic glycolysis predominates owing to limited oxygen availability. In this zone, glycolysis is essential for ATP production, which supports vital cellular functions, such as ion homeostasis and osmotic regulation via ATP-dependent ion pumps [132]. Additionally, glucose is a key precursor for the synthesis of ECM components [145], including proteoglycans and glycosaminoglycans, which are crucial for maintaining the cartilage structure and function under mechanical stress [111,114,115]. A decrease in glucose availability disrupts both energy production and ECM biosynthesis, weakening the cartilage matrix and impairing its ability to resist compressive force. This metabolic vulnerability is most pronounced in the deep zone, where nutrient transport is restricted [31,32,33,35,36,152], exacerbating the risk of cartilage degeneration and contributing to OA development. These findings underscore the critical role of glucose in maintaining cartilage homeostasis and highlight the need to optimize the nutrient supply to preserve cartilage integrity and reduce the risk of developing degenerative joint diseases. In this context, synovial clearance of vital substances (e.g., glucose, oxygen, and ions) is a crucial mechanism that regulates nutrient availability and may play a significant role in the development of OA. The diffusion of small molecules, such as glucose, through cartilage is a slow process, with an approximate velocity of 50 μm per minute [31,32,33,36,152]. This limited diffusion rate is further compounded by the potential role of synovial fluid clearance in thick cartilage malnutrition. The synovial fluid in the joint cavity is estimated to have a clearance time of approximately one hour [34,154]. This limited time window highlights the challenge of essential nutrients for the effective penetration and nourishment of articular cartilage. The transport of nutrients to chondrocytes heavily relies on diffusion, and the efficiency of this process is influenced by the agitation of synovial fluid [31]. During periods of reduced joint movement, such as rest, the permeability of cartilage decreases, and stagnant fluid can hinder the transport of vital nutrients, resulting in inadequate nourishment of the deeper layers of cartilage. If malnutrition persists, it can lead to chondrocyte death through apoptosis, making the cartilage more susceptible to matrix degradation and further apoptosis. This vicious circle is believed to play a critical role in OA pathogenesis [155].

Joint movement is critical for the maintenance of chondrocyte function and homeostasis. Nevertheless, the consequences of sustained or prolonged sedentary behavior on the integrity of hyaline cartilage are still not well understood. It is proposed that this prevalent behavior may contribute to a specific molecular endotype, attributed to the detrimental effects of the ionic environment on large macromolecules within the context of “unstirred cartilage”. This could potentially represent a very early stage and distinct phenomenon in knee and hip OA as a manifestation of decreased cellular function in “sedentary-thick cartilage”. Ionic equilibria play a crucial role in shaping the conformation and interactions of macromolecules within cartilage, influencing their interplay with the bulk water pool. This, in turn, has a profound impact on ECM organization. In sedentary adults, early OA is hypothesized to originate from a stagnant thin film of synovial fluid adhering to the rugose surface of cartilage. Under vigorous mixing, the stagnant liquid film remains on the order of 10–100 µm; however, in the absence of fluid motion, it can thicken to the millimeter scale [148,149,153,156]. This film acts as a barrier, restricting the transport of essential nutrients and facilitating the accumulation of waste products within deeper layers of the cartilage. As a result, the altered microenvironment surrounding the chondrocytes has a significant effect on cellular metabolism. The underlying mechanism may involve an imbalance between the regulation of cartilage matrix glycoprotein expression and production of pro-inflammatory and proteolytic proteins. The anti-anabolic component of this imbalance is specifically attributed to the reduced turnover rate of keratan and chondroitin sulfate molecules as well as the compromised maintenance of certain low-molecular-weight non-collagenous bond glycoproteins. These compounds are essential for preserving the integrity of glycosaminoglycan chains, proteoglycan networks, hyaluronic acid, and collagen fibers, which are highly susceptible to disorganization [157]. Proteolysis of proteoglycan-hyaluronic acid and collagen fiber attachments is mediated by the activities of aggrecanase (ADAMTS) and matrix metalloproteinase (MMP) [158,159]. This process is often triggered by the accumulation of free radicals, oxidizing species, or waste products, which can initiate the degradation of these attachments. Metabolic imbalance disrupts the regulation of cartilage matrix glycoproteins, leading to the production of proinflammatory cytokines and proteolytic enzymes. Deterioration of the integrity of glycosaminoglycans, proteoglycans, hyaluronan, and collagen compounds exacerbates this condition [18]. Although cartilage possesses mechanisms for self-repair through protein binding, its capacity for complete restoration is limited [160,161]. Heinemeier et al. [162] used carbon-14 bomb-pulse dating to demonstrate that collagen in adult human tibial plateau cartilage is virtually permanent—with negligible turnover after skeletal maturity and no measurable increase in replacement rates irrespective of osteoarthritic changes—highlighting its limited ability to self-repair. The slow turnover of collagen in adult humans contributes to the accumulation of non-enzymatic post-translational modifications, hindering repair and leading to established OA [163]. Thus, it is plausible that this early-stage OA may play a critical role in the very early stages of the osteoarthritic process, particularly just before the onset of structural and functional alterations in the collagen fibers. Damaged collagen cannot be fully repaired [162], thereby perpetuating OA progression. Early changes in the articular surface, such as fibrillation, cracking, and thinning, are often imperceptible because of the absence of nerve endings in hyaline cartilage. This subtle, asymptomatic damage can progress gradually, potentially leading to joint inflammation and structural modifications. Symptoms typically become evident only when the damage affects the surrounding joint structures and underlying bone, ultimately resulting in the diagnosis of established OA through radiological imaging. At this advanced stage, the disease can severely impair joint function and mobility, leading to significant disability and a reduced quality of life. In cases where conservative treatment is ineffective, total joint replacement may be required to restore function and provide relief from chronic pain. This highlights the significance of addressing OA in the early stages of therapeutic intervention to preserve cartilage health and effectively mitigate disease progression.

However, the elusive nature of the earliest phase of OA, which challenges conventional understanding, demands a paradigm shift in its approach, particularly in preserving robust cartilage structures. Detection of this phase may require advanced imaging methods and precise biochemical or ultrastructural markers [9,164,165,166,167]. In the context of OA in the early stages, the dGEMRIC (Delayed Gadolinium-Enhanced MRI of Cartilage) technique stands out for precisely assessing glycosaminoglycan content in cartilage and detecting reduced glycosaminoglycan concentrations indicative of initial degeneration [168]. Although T2 mapping primarily detects collagen fiber changes in established OA, it could offer insights into the early OA stages by revealing decreased water content and potential abnormalities [166]. Sodium MR, in conjunction with proton MRI, provides a direct and noninvasive method for quantifying glycosaminoglycan content and fixed charge density (FCD) in cartilage, particularly in young adults with ‘intact’ and thick osteochondral tissue [167]. This technique shows promise as an imaging biomarker to monitor glycosaminoglycan loss and cartilage degradation over time in healthy individuals. However, challenges in sodium concentration quantification persist and require further advancement for seamless clinical translation in the early detection of metabolic imbalances preceding established OA. As we enter an era of regenerative medicine, understanding the impact of stagnant films becomes imperative. This understanding is not only essential for the success of advanced techniques for repairing articular cartilage but also for improving pharmacological treatment in research laboratories, clinical studies, and practical clinical applications. Drug efficacy in targeting cartilage may be compromised by diffusion barriers due to stagnant fluid layers [169]. Importantly, joint motion may serve as a crucial factor in enhancing the efficacy of chondroprotective agents, potentially transforming unsuccessful compounds into effective therapeutics for cartilage preservation. Therefore, combining pharmacological interventions with physical therapy has the potential to enhance therapeutic effectiveness significantly. However, this area of research remains in its infancy. The knowledge derived from ongoing research will be critical in designing future clinical trials to evaluate both conventional and emerging intra-cartilage treatments, including the controversial chondroprotective agents glucosamine and chondroitin sulfate [170,171].

## 12. Mechanistic Drivers of Sedentary Lifestyle-Induced Osteoarthritis: Unstirred Layers, Nutrient Deprivation, and the Emerging *Sedenthrosis* Phenotype

OA is too often mislabeled as merely *degenerative arthritis* a term that fails to capture the active biological processes underlying its progression. In reality, OA is not simply the outcome of passive wear-and-tear; it results from a dual interplay between the degeneration of hyaline cartilage and attempted regeneration of subchondral bone [16,78,79,80,81,82,83,84]. Inflammatory cytokines, such as tumor necrosis factor-alpha (TNF-α) and interleukin-1 beta (IL-1β), activate chondrocytes to produce matrix-degrading enzymes, such as MMPs and ADAMTS, which are instrumental in breaking down the ECM. The limiting depth of hyaline cartilage, the presence of unstirred layers, and nutrient deprivation in the development and progression of knee and/or hip OA can have significant and far-reaching implications. Characterized by its avascular, non-insulin-sensitive, and glycolytic nature, cartilage primarily relies on glucose diffusion for chondrocyte homeostasis and ECM integrity [144]. In this regard, Maroudas et al. [31] found that the joint cartilage exhibits direction-dependent diffusion resistance caused by the constrictive flow of stagnant fluid films on its surface. This effect hinders the normal daily exchange of substances within the cartilage, leading to reduced mass transport and tissue dehydration. In young, healthy individuals, articular cartilage typically retains sufficient thickness to be affected by nutrient diffusion limitations, particularly in the deeper zones where stagnant liquid films and poor synovial fluid agitation restrict the supply of vital nutrients, such as glucose and oxygen [31,32]. This phenomenon can lead to metabolic stress in deep-layer chondrocytes, impairing their ability to maintain ECM and initiating early cartilage degeneration. However, as the cartilage progressively loses thickness owing to nutrient deprivation, the limiting depth becomes less of a risk factor. Paradoxically, although thinner cartilage may now receive adequate nutrient diffusion because the unstirred layers are less influential, this joint tissue faces greater risk from mechanical factors. Thinning compromises its ability to withstand cyclic loading and mechanical stress and results in the loss of a significant portion of its tissue responsible for cushioning impacts. As a result, hyaline cartilage, once vulnerable to nutrient deprivation, becomes increasingly susceptible to early OA owing to mechanical degradation, triggering a vicious cycle in which damage to the superficial structure exacerbates bulk nutrient deficiency and accelerates further mechanical breakdown [31,36]. This underscores the importance of timely intervention to preserve cartilage thickness and prevent transition from metabolic to mechanical failure.

In healthy cartilage, especially in joints such as the knee and hip, nutrient delivery from synovial fluid is essential for maintaining the viability of chondrocytes [31,34,35,37,128]. However, under conditions of limited motion such as prevalent modern sedentary behaviors, a stagnant synovial fluid layer can develop at the cartilage surface, increasing its effective thickness and reducing the availability of nutrients to chondrocytes located deeper within the cartilage. As previously mentioned, this stagnant liquid film acts as an additional barrier to diffusion [31,34], significantly limiting the delivery of glucose and oxygen, which is critical for sustaining chondrocyte metabolism. As glucose diffusion becomes restricted, the chondrocytes in the deeper zones of cartilage experience metabolic stress, leading to a reduction in ATP production, impaired cellular function, and diminished ECM biosynthesis [131,132,144,145]. This metabolic imbalance accelerates the degradation of ECM components such as proteoglycans and collagen, which are vital for cartilage structural integrity. Insufficient nutrient delivery to cartilage exacerbates its vulnerability to mechanical stress, initiating a cascade of degeneration that is characteristic of the early stages of OA. The increased cartilage thickness plays a pivotal role in joint function by providing a larger reservoir for interstitial fluid, allowing it to dissipate mechanical loads laterally. This process reduces localized hydrostatic pressure and maintains the force distributed across a larger joint surface area, thus keeping joint stress within physiological limits and preserving cartilage integrity [14,122]. However, in early OA, disuse atrophy leads to chondropenia—marked thinning of hyaline cartilage—which compromises its capacity to withstand mechanical loads. Thinner cartilage limits the availability of interstitial fluid required to buffer mechanical impacts, impairing the hydrostatic pressurization mechanism that protects the tissue. This results in elevated joint surface stresses and localized overload, which exacerbate matrix degradation. Moriyama et al. [172] further showed that immobilization after spinal cord injury causes cartilage thinning to be driven predominantly by loss of the calcified cartilage layer—off-loaded regions lost roughly 30% of thickness in this zone—accompanied by proteoglycan depletion in the non-calcified layer, underscoring the pivotal role of the calcified zone in maintaining cartilage integrity. These findings highlight how the disruption of calcified cartilage may set the stage for aberrant ossification and progressive calcification within the tissue.

**Figure 6:**
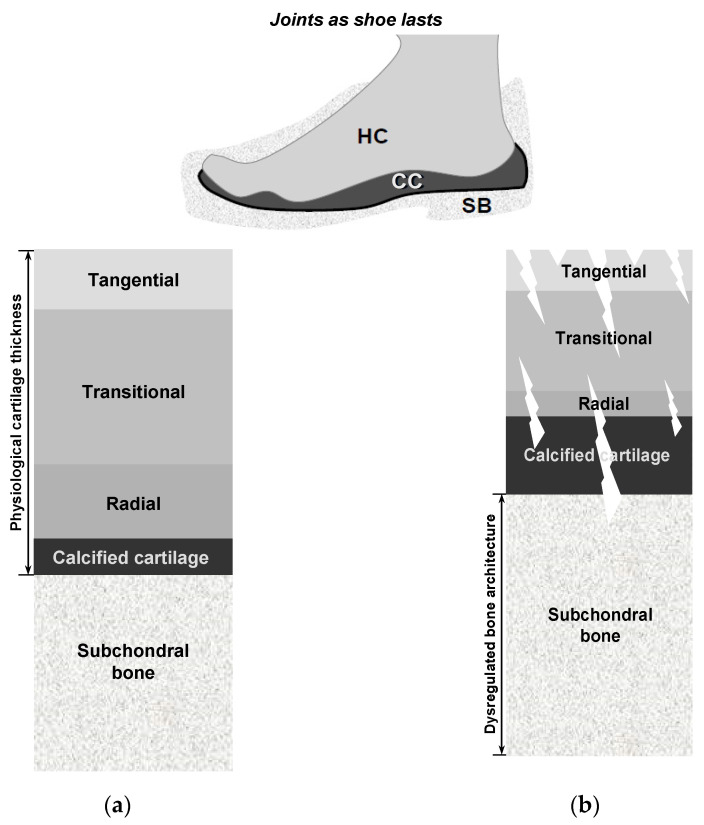
Schematic representation of the structural differences between healthy and degenerated osteochondral units. (**a**) A cross-sectional view of healthy nonmineralized and mineralized cartilage illustrating distinct layers—tangential, transitional, radial, and calcified zones—along with the underlying subchondral bone, all depicted under optimal conditions. This well-preserved cartilage exhibits appropriate thickness, maintaining its functional integrity and effective load distribution during joint movement. (**b**) Cross-sectional view of degenerated cartilage, demonstrating significant thinning, development of fissures, deterioration of deep hyaline cartilage layers, and thickening of the calcified cartilage associated with endochondral ossification. The subchondral bone exhibits pathological thickening, sclerosis, angiogenesis, bone cyst and osteophyte formation, and cracking—characteristic features of advanced OA. These changes reflect compromised structural integrity and impaired mechanical function of the bone-cartilage unit, thereby emphasizing the progression of joint degeneration. The ability of subchondral bone to absorb mechanical shock decreases over time, resulting in increased stress on the remaining uncalcified cartilage. Early intervention is essential for preserving joint health, especially before the thinning of uncalcified cartilage reaches a stage where depth limitations are no longer significant and permanent structural damage occurs. Illustration not to scale; adapted from [80].

In response to the demands of bipedal evolution, humans likely evolved thicker cartilage in weight-bearing joints [69], such as the knee and hip, which enabled hominin ancestors to engage in high-impact activities, such as running and long-distance walking, often exceeding 20 km per day [24]. However, this unproven evolutionary hypothesis raises significant concerns, suggesting potential nutrient deprivation in thick cartilage, which may exacerbate degeneration and increase susceptibility to OA. Nutrient deprivation disrupts chondrocyte function, leading to a reduction in ECM synthesis and triggering cell death via apoptosis [35]. As chondrocytes die and the matrix degrades, cartilage loses its structural and functional integrity. As the ECM deteriorates, chondrocytes shift to a hypertrophic phenotype, synthesizing a fibrocartilage-like matrix that lacks the load-bearing properties of a healthy hyaline cartilage. This shift triggers endochondral ossification, wherein the deep cartilage is gradually replaced by bone [16,78,79,80,81,83]. Hypertrophic chondrocytes undergo apoptosis, allowing for the invasion of blood vessels into previously avascular cartilage regions. This invasion, combined with the calcification of the cartilage matrix, drives the formation of osteophytes and contributes to thickening of the subchondral bone, as schematically shown in Figure 6. Simultaneously, the balance between osteoblast and osteoclast activity in the subchondral bone is disrupted, leading to increased bone turnover and formation of subchondral sclerosis. As the subchondral bone becomes denser and harder, it loses its ability to effectively absorb mechanical forces, further increasing the mechanical stress placed on the overlying cartilage. This accelerates cartilage degeneration, perpetuating a destructive cycle of joint damage, in which inflammation of the synovial membrane (synovitis) plays a key role. The inflamed synovium releases proinflammatory cytokines and catabolic enzymes, further degrading the cartilage matrix, impairing joint lubrication, and increasing friction within the joint [13]. Over time, this chronic inflammation not only worsens cartilage loss but also leads to fibrosis and thickening of the synovial membrane, contributing to joint stiffness and pain and ultimately driving the progression of OA.

As OA progresses, the calcified layer of cartilage begins to fissure and crack, allowing deeper penetration of the vascularized subchondral bone. This leads to the infiltration of molecules from the vasculature, triggering inflammation and fluid accumulation (edema) within the osteochondral unit, commonly known as the bone-cartilage unit [173,174]. These pathological processes further disrupt the biomechanical integrity of the joint and lead to the characteristic pain associated with advanced OA. At this stage, the metabolic imbalance between cartilage and bone becomes more pronounced. The altered phenotype of osteoblasts contributes to abnormal bone formation, whereas hypertrophic chondrocytes drive ECM mineralization, leading to chondrocalcinosis. As a result, calcified cartilage no longer functions effectively as a load-bearing tissue, and the joint begins to collapse structurally. The subchondral bone becomes stiffer, creating patterns of abnormal joint stress that further contribute to mechanical failure. Progressive loss of cartilage leads to the exposure of the subchondral bone, causing severe damage to the joint. This ultimately results in the hallmark symptoms of OA, including pain, stiffness, and loss of joint mobility, culminating in the need for surgical intervention, such as total joint replacement, in advanced stages of the disease.

In Figure 6a, distinct layers of healthy articular cartilage are shown, including the tangential, transitional, and radial zones. These layers are in an optimal condition, preserving the functional integrity of the cartilage [114,121]. The tangential zone, with its horizontally aligned collagen fibers, provides resistance to shear forces, whereas the transitional zone offers a bridge between the tangential and radial zones, absorbing mechanical stresses. The radial zone, characterized by vertically aligned collagen fibers, plays a critical role in cushioning compressive loads. Below the cartilage, the subchondral bone is depicted in a healthy, well-maintained state, showing no signs of abnormal thickening or damage. This figure represents the archetype of thick, normal cartilage in load-bearing joints, such as the knee and hip, where the balance of mechanical and metabolic processes maintains joint function. In contrast, Figure 6b illustrates the pathological changes characteristic of advanced OA. The once thick cartilage is now significantly thinned owing to the loss of the deep radial zone, a consequence of endochondral ossification [78,79,80,81,83]. This process, triggered by nutrient deprivation and altered mechanobiological forces, leads to duplication of the tidemark, along with subsequent calcification and ossification of the cartilage. The subchondral bone, which appears intact in Figure 6a, is now pathologically thickened, indicating sclerosis—a hallmark of OA progression. Angiogenesis or invasion of blood vessels into cartilage is also evident in the degenerated osteochondral unit, contributing to its deterioration [173,174]. Additionally, bone cracking is observed in the subchondral layer, which results from the increased mechanical stress and impaired shock absorption capability of the cartilage. These cracks compromise the structural integrity of the bone and contribute to further joint instability. The combination of these pathological changes—including cartilage thinning, subchondral bone thickening, angiogenesis, and bone cracking—leads to a destructive cycle of joint degeneration, ultimately resulting in the collapse of the bone-cartilage unit and complete loss of functional joint integrity.

Although the long-term significance of the detrimental effects of nutrient deprivation in joint cartilage regarding OA in later life remains speculative, this cascade of events may contribute to its onset in young adults [4,5], which is attributed to the prevalent sedentary behavior characteristic of contemporary lifestyles [29]. As noted by Lieberman et al. [21], the prevalence of knee OA has doubled since the mid-20^th^ century, underscoring a significant public health challenge as society transitions into a post-industrial era. This concerning trend may be closely associated with significant changes in the cellular metabolism of hyaline cartilage, influenced by the prevalent sedentary lifestyle. This sharp increase is not an isolated incident; it reflects a broader and alarming trend in incidence rates, with projections from Schwarzkopf et al. [8], indicating a dramatic increase in the demand for total knee and hip replacements by 2040–2060. As mentioned earlier, dynamic agitation of synovial fluid through regular physical activity plays a critical role in reducing the impact of unstirred layers [31,33,34]. Low-impact physical activities, such as cycling or walking, can help maintain nutrient delivery to the cartilage by reducing stagnant fluid film and enhancing nutrient diffusion. This is particularly important in preventing OA in high-load joints like the knee and hip, where cartilage thickness and metabolic demands are significant [35]. Carter et al. [78] provided key insights into the mechanobiology of articular cartilage, highlighting the intricate relationship between mechanical loading, cartilage development, and degeneration. Their work underscores how biomechanical factors, such as hydrostatic pressure and tissue deformation, regulate cartilage homeostasis and contribute to its deterioration under pathological conditions. Their findings suggest a mechanobiological feedback loop, in which cartilage degeneration and subchondral bone stiffening perpetuate joint deterioration, offering a mechanistic framework for understanding how OA progresses as a result of nutrient deprivation and altered mechanical loading. Xiao et al. [82] provided further insights into the complex interplay between cartilage degradation and subchondral bone remodeling in OA, drawing parallels between osteochondral remodeling in OA and the process of endochondral ossification observed in growth plates. Their work suggests that similar to endochondral ossification, cartilage thinning and ECM degradation in OA are accompanied by increased subchondral bone remodeling, where chondrocytes undergo hypertrophic differentiation, contributing to calcification and ossification within the cartilage. This process not only accelerates the loss of cartilage, but also promotes structural changes in the subchondral bone, further exacerbating joint degeneration. These findings support the mechanobiological feedback loop proposed by Carter et al. [78], where cartilage thinning and subchondral bone advancement fuel each other and drive the progression of OA through a cycle of biomechanical and metabolic disruptions.

Further research is essential to fully elucidate the potential connection between nutrient deprivation in the deep zones of the knee and hip cartilage, particularly in modern sedentary lifestyles, and the subsequent progression to cartilage ossification. This nutrient deficiency, exacerbated by stagnant synovial fluid and reduced physical activity, may be a key factor in the alarming increase in OA prevalence in recent years [4,5,21]. Understanding this early stage mechanism could also provide insights into the accelerated degeneration of cartilage, ultimately leading to ossification and joint failure [16,78,79,80,81,83]. The increasing prevalence of OA, coupled with concerns about the long-term health of the knee and hip joints [8], highlights the urgent need for proactive interventions that address early metabolic disturbances associated with cartilage degeneration. Such strategies are essential not only to mitigate the onset of OA, but also to alleviate the significant burden of its related comorbidities [175], ultimately enhancing the quality of life of individuals at risk. Addressing this issue is imperative as it represents a significant public health challenge that, without intervention, threatens to impact global mobility and quality of life for future generations. Therefore, a comprehensive approach is urgently needed to overcome this emerging health crisis. Focusing on early detection, regular joint-friendly exercises and targeted therapeutic interventions are critical for maintaining cartilage health in young individuals with intact full-thickness cartilage. This integrated approach has the potential to enhance nutrient availability and optimize the removal of metabolic waste, significantly reducing the long-term risk of knee and hip OA and effectively addressing a critical public health challenge.

## 13. *Sedenthrosis*: Medical Condition, Social Challenge—or Both?

In the context of an escalating epidemiological crisis of OA, *sedenthrosis*—OA driven by chronic inactivity—emerges not only as a cartilage degenerative disease but also as the clinical endpoint of a societal failure to prioritize movement. This reflects pervasive disruptions in lifestyle behaviors, cultural values, and socioeconomic structures of contemporary life. The increasing prevalence of knee and hip OA—particularly the *sedenthrosis* phenotypes—should therefore be understood as a symptom of how society organizes daily life, mobility, and work, and not solely as a disease of aging or biomechanics. Against this backdrop, OA emerges as a consequence of modern sedentary lifestyles [21]. Unless we confront these root causes, all the pills, injections, and surgeries in the world will only treat the symptoms and never address the origin of the epidemic. Particularly, hip and knee arthroplasty, though effective for end-stage disease, is constrained by soaring costs, protracted waitlists, and limited specialist capacity, underscoring the unsustainability of a purely biomedical response to a population-level epidemic [8]. Remaining inactive is no longer a viable strategy. Healthcare systems simply cannot sustain the clinical and economic burden generated by an epidemic of inactivity-driven OA. Quantitative analyses forecast nearly one billion people living with OA by 2050 [1], driving healthcare expenditures—currently dominated by curative care, such as hospitalizations, specialist consultations, surgeries, and chronic pain pharmacotherapy—to unsustainable levels. In contrast, preventive spending occupies a small fraction of budgets despite clear evidence that investments in active urban design, workplace movement mandates, and behavior change incentives yield multi-fold downstream savings. In this context, “doing nothing” might be the costliest option: every euro or dollar diverted solely to downstream treatments is a euro not spent on the upstream solutions capable of halting disease progression at its source. Emerging evidence from large-scale community interventions, such as the GLA:D initiative [176], demonstrates that proactive approaches involving structured exercise and education programs not only improve patient outcomes but also exhibit favorable cost-effectiveness profiles, offering a sustainable path forward amid the growing burden of OA and highlighting prevention as both a clinical and economic imperative.

**Figure 7:**
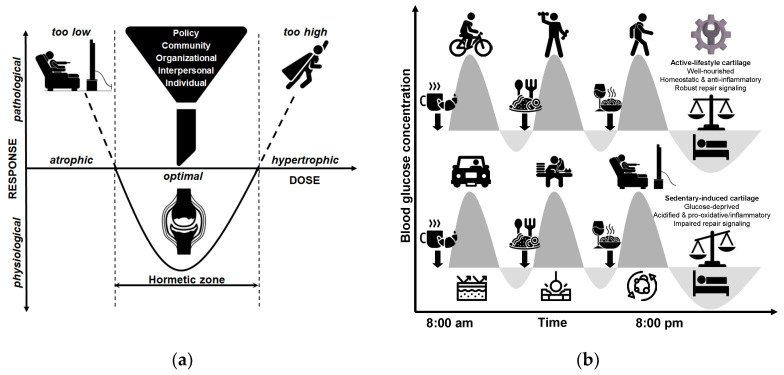
(**a**) The hormetic dose–response of articular cartilage to biomechanical stress defines three critical zones: understimulation, optimal stimulation, and overstimulation. In the understimulation region—often summarized by the “use it or lose it” principle—insufficient (subthreshold) joint motion fails to generate adequate synovial fluid stirring and convection, impairing nutrient diffusion and leading to matrix atrophy and cartilage degeneration. In contrast, the optimal mechanical stimulus (adaptive) window engages physiological stress that preserves well-nourished cartilage cells, activates homeostatic and anti-inflammatory signaling cascades, suppresses catabolic mediators (e.g., MMP-3, MMP-13), and drives robust anabolic pathways (e.g., TGF-β, IGF-1) for effective ECM repair. At the opposite extreme, the over-stimulation (maladaptive) region—aligned with the “wear and tear” theory—occurs when excessive mechanical stress overwhelms chondroprotective mechanisms, provokes pro-inflammatory cytokine release (e.g., IL-1β, TNF-α) and oxidative damage, and precipitates hypertrophic changes and osteophyte formation. Maintaining joint motion within this narrow hormetic zone is essential to bridge the clinical “care gap” between disuse atrophy and cumulative cartilage damage—and, as illustrated by “the socioecological funnel”, depends on a supportive framework of multifaceted determinants of health, including the macrosystem (public policy and community structures), mesosystem (institutional/organizational and social/interpersonal networks), and microsystem (individual level), which together generate and sustain the optimal mechanical stimulus. (**b**) Translational lifestyle strategy for chondroprotection via postprandial–mechanical coupling: Active lifestyle paradigm (upper panel): synchronizing each postprandial glycemic peak with targeted joint loading (e.g., cyclic compression during cycling, resistance exercise, ambulation) enhances synovial fluid convection, improves nutrient and oxygen diffusion across the cartilage boundary layer, augments ATP-dependent repair processes, and reinforces anti-inflammatory signaling. Sedentary paradigm (lower panel): identical glucose excursions followed by prolonged inactivity (motorized transport, desk work, screen time) lead to stagnant synovial exchange, lactate accumulation, oxidative stress, and increased MMP activity, promoting a degenerative cartilage phenotype. Note: Synovial fluid is treated as a well-mixed compartment; in vivo boundary layer dynamics from transient cytokine, hormonal, and metabolite gradients may further modulate nutrient transport.

Effectively addressing the *sedenthrosis* epidemic may require an imminent transition from reactive to proactive joint health strategies. In the femoral joints of the hip and knee, articular cartilage—a tissue intrinsically kinetic in nature—relies on a finely narrow hormetic range of mechanical stimuli; too little stress leads to atrophic degeneration, whereas too much stress leads to hypertrophic damage and structural deterioration of the cartilage. Figure 7a depicts this classic hormetic dose–response curve (inverted U-shaped) with three key zones [177]: (1) an under-dose region where mechanical deficiency causes maladaptive thinning; (2) a central chondroprotective zone of optimal homeostatic benefit; and (3) an over-dose region where excessive stress triggers pathological hypertrophy and osteophyte formation. The threshold dose marks the transition into homeostatic territory—underscoring that “less can be more, but not so little, and certainly not too much”.

However, modern movement patterns often polarize at opposing extremes [29]. The prototypical “couch potato” remains virtually inactive, rooted in the under-dose region. Conversely, the so-called “active couch potato”—including “Weekend Warriors” and “Ironmen”—engage in rigorous endurance training cycles capable of inducing cartilage overload and microtrauma (wear-and-tear), yet otherwise lead a “gym-sedentary” lifestyle, sitting for eight or more hours daily and ultimately reverting to the same under-dose domain outside their workouts. Despite their radical differences in intention and identity, both profiles share a common defect: prolonged sedentary intervals that deprive cartilage of the periodic movement necessary to reside within the hormetic (chondroprotective) zone (Figure 7a). Addressing *sedenthrosis* thus demands not only targeted exercise prescriptions but also holistic, system-level interventions to reshape daily movement and rest cycles, thereby ensuring sustained joint-protective mechanical stimuli.

The rising prevalence of overweight and obesity in industrialized societies exacerbates the consequences of sedentary behavior on joint health. Although physical inactivity is a primary driver of increased adiposity, obesity contributes to OA pathogenesis through both mechanical and metabolic pathways [4,5]. Excess body weight imposes elevated biomechanical loads on hip and knee articulations, accelerating ECM wear, while adipose-derived mediators—particularly leptin—have been shown to induce cartilage catabolism and promote synovial inflammation [11]. Within the *sedenthrosis* paradigm, obesity may further compromise articular cartilage nourishment by diminishing matrix permeability, a phenomenon demonstrated by the inverse correlation between compressive loading and solute diffusion in the diarthrodial cartilage [133]. Thus, overweight and obese individuals potentially experience an exacerbated insult, wherein intensified mechanical stress synergizes with constrained nutrient delivery during periods of joint movement. This dual burden not only predisposes load-bearing joints to accelerated degenerative changes but also underscores the urgency of integrated interventions that address both biomechanical loading and metabolic regulation in sedentary, overweight populations.

Based on the previously described hormetic threshold, Figure 7b illustrates this emergent concept in terms of metabolic synergy. The upper panel depicts three postprandial blood glucose peaks—after breakfast, lunch, and dinner—each followed by timely joint movement (e.g., active commuting, lunchtime exercise, after-dinner walking), potentially enhancing nutrient delivery, accelerating glucose diffusion into synovial fluid, and sustaining anti-inflammatory and anabolic pathways [37,144,145]. In theory, this “active lifestyle cartilage” phenotype is characterized by a well-nourished matrix, homeostatic signaling, and robust repair capacity. In contrast, the lower panel shows identical glucose peaks followed by habitual sedentary behavior (car commuting, prolonged desk confinement, and passive evening routines), in which the absence of mechanical stimuli compromises nutrient exchange, fosters acidification and oxidative stress, and leads to a “sedentary-induced cartilage” state marked by glucose deprivation, proinflammatory signaling, and impaired repair processes. Critically, this prolonged inactivity also disrupts the biomechanical “pump” mediated by hyaluronan: without regular flexion–extension cycles, the Donnan osmotic back-pressure and viscosity contributions of hyaluronic acid diminish, fluid turnover slows, and cartilage loses its dynamic reservoir of lubrication and nutrients [178]. To address these challenges, the novel CADENCE framework—a circadian-synchronized, four-phase intervention model—holds promise as a biologically informed scaffold for aligning timely joint motion with metabolic rhythms, potentially enhancing nutrient transport, optimizing chondroprotective therapies, preserving homeostatic balance, and mitigating both mechanical under-stimulation and excessive stress on cartilage tissues [171].

Nevertheless, the root of the *sedenthrosis* epidemic presumably lies in the social determinants of health, which fundamentally shape the ability of individuals to access and engage in mechanical stimuli essential for cartilage homeostasis. Factors such as socioeconomic status, quality of education, income stability, social support, healthcare access, health literacy, mental health, environmental safety, economic inequality, and spread of misinformation collectively govern whether one can achieve the optimal mechanical dosing required by joint cartilage [179,180,181]. When these determinants fail, the cartilage becomes chronically underloaded or subjected to excessive stress (Figure 7a). Sedentary occupations, such as office work, driving, and cashiering, can be understood, from a conceptual standpoint, as falling on the underdose limb of the hormetic curve, depriving cartilage of necessary cyclic loading. In contrast, it is plausible that repetitive high-impact activities—common in construction, agriculture, mining, healthcare patient handling, and certain sports—may exceed the adaptive threshold [177], inducing hypertrophic pathology and diminishing the protective effects afforded by the optimal chondroprotective zone.

Bridging this deficiency requires at least two convergent strategies. Clinically, precision dosing of mechanical stimuli through personalized physiotherapy protocols and wearable-driven targets calibrated to the PALs of individuals during ADLs can maintain patients within the hormetic sweet spot. Simultaneously, transformative public health and occupational policies must reshape environments and incentives: sit–stand mandates, micro-break scheduling, rotational tasks, active urban design (walkable streets, protected bike lanes, accessible green spaces), and ergonomic assistive devices in high-risk industries. Equally imperative is the creation of a compelling, human-centered narrative—crafted by social scientists, humanists, and communication experts—to reframe the movement as both a personal imperative and a shared societal value, countering the infodemic and rebuilding collective motivation. Crucially, reshaping behavior demands more than data—it requires a compelling narrative that unites reason and emotion.

Building on that narrative, *sedenthrosis* will not yield isolated medical interventions; it is a social disease rooted in policy neglect and a narrative vacuum, demanding a shift from reactive treatment to proactive transformation. Accordingly, structural reforms must be paralleled by a paradigm shift in societal and cultural perceptions of OA—reimagining it from an inexorable hallmark of aging into a modifiable condition responsive to prevention, resilience, and self-management. Nationwide campaigns such as “Sit Less, Move More”, “Every Step Counts”, or “Be Active, Feel Good”—embedded in school curricula and community programs—can shift social norms to prioritize regular standing and walking. Clinical practice should embrace “social prescriptions”, whereby physicians refer patients to community exercise initiatives, urban planning consultations, or policy advocacy platforms alongside conventional pain management. Digital health platforms can synthesize wearable-derived activity data with patient-reported outcomes, enabling personalized intervention packages and real-time monitoring of both clinical progress and environmental uptake. Such an integrative paradigm—integrating medical therapies with ambitious public health policies and cultural change—may constitute the only sustainable path forward.

Putatively, *sedenthrosis* will yield only when we recognize it as both a medical condition and a social challenge. This demands the convergence of precise biomechanical dosing, optimized metabolic coordination, and sweeping socioecological reforms. By mapping cartilage health onto the hormetic curve [177], closing the deficit in protective mechanical stimuli through environmental and policy shifts [179,180,181], and addressing the upstream social determinants that govern our daily mechanical “dose”, we can transform OA management from reactive to proactive—ensuring that joint tissues receive the stress they need to thrive within the social contexts that sustain healthy movement. Through this comprehensive strategy, by realigning clinical practice with preemptive public health action and narrative transformation, we can restore the kinetic equilibrium essential for cartilage homeostasis and, in doing so, cultivate healthier and more resilient communities. Evidence-based and successful initiatives such as GLA:D [176] highlight how early movement-based interventions can effectively shift the course of OA at the population level while contributing to the long-term sustainability of health systems.

## 14. Limitations, Challenges and Future Perspectives

Despite providing valuable insights into the critical role of cartilage thickness in OA development and progression, this study was constrained by several methodological and conceptual limitations that warrant careful consideration. Primarily, it adopts a cartilage-centric perspective, focusing exclusively on hyaline cartilage health and function, which may overlook the contributions of other joint tissues, such as the subchondral bone, synovium, ligaments, and menisci [10,39]. Effectively managing OA requires more than merely caring for the cartilage, as simplistic and reductionist strategies have been proven insufficient. It demands a comprehensive approach that addresses all potential risk factors, recognizing OA as a complex disease that affects not only the entire articular organ but also the systemic physiology [10,11,12]. The reliance on in vitro and ex vivo models restricts the extrapolation of the findings to the complex in vivo environment, where systemic factors—ranging from metabolic status to inflammatory mediators—play pivotal roles. Additionally, the assessment of physical activity/exercise recommendations did not account for patient heterogeneity in terms of comorbidities or biomechanical loading patterns, potentially limiting clinical applicability. Therefore, future studies should embrace a holistic, person-centered care that integrates multifactorial risk factors, including obesity, muscle weakness, joint laxity, injury history, and genetic predisposition, to develop tailored evidence-based interventions that adhere to the principle of *primum non nocere*, or simply, “first, do no harm”.

Although cutting-edge computational and ex vivo cartilage models have significantly advanced our understanding of solute diffusion and mechanotransduction, they have not been validated in vivo, where the interplay of physiological loading, synovial fluid dynamics, and zonal heterogeneity critically shapes nutrient delivery and cell survival. To date, no study has quantitatively assessed nutrient transport or directly correlated chondrocyte viability with cartilage depth (limiting depth) in native human tissue under physiological conditions. Addressing this deficiency will require the development of minimally invasive in vivo techniques, such as depth-resolved PET/MRI tracers for nutrient analogs, fiber-optic microprobes for interstitial oxygen and pH measurements, or novel contrast-enhanced ultrasound protocols, rigorously benchmarked against gold-standard ex vivo assays. Such efforts must be supported by robust ethical frameworks and standardized risk-benefit analyses to ensure patient safety while facilitating the translation of preclinical findings into an accurate, zonally informed map of human cartilage health and degeneration.

There are further inherent limitations to this proposal. Importantly, these recommendations lack validation in preventive medicine, and to the best of my knowledge, there are no evidence-based guidelines specifically tailored for this purpose. The presence of relatively thick articular cartilage in the tibiofemoral, patellofemoral, and coxofemoral joints—likely a derived adaptation to the increased loading demands of bipedal locomotion—may constitute an inherent, non-modifiable risk factor, as its structural characteristics, regional thickness variations, and biomechanical properties reflect evolutionary optimization for sustained weight-bearing rather than reparative capacity [69]. This highlights the need for targeted prevention research and the design of intervention programs aimed at optimizing nutrient supply within the deep zone of thick cartilage in load-bearing joints. Traditional WHO recommendations primarily target the prevention of age-related non-communicable diseases, sarcopenia, and osteopenia; however, their specific effectiveness in preventing disuse-induced cartilage atrophy, particularly regarding tissue thinning, compromised nutrient transport through unstirred layers, and depth-dependent degeneration, remains unclear. These observations underscore the need for further research to elucidate the pathophysiological mechanisms of age-associated chondropenia [182], particularly its role in initiating degenerative cartilage disease, termed herein as *sedenthrosis* in the context of physical inactivity. Multifaceted engagement emphasizes the significance of preventive healthcare practices in shaping a holistic and well-informed approach to physical education.

Despite introducing *sedenthrosis* to characterize inactivity-induced cartilage degeneration in the hip and knee, our study remains narrowly focused on nutritional determinants in these two key weight-bearing synovial joints, neglecting several important aspects. Notably, I did not examine how cartilage thickness variability in less-studied upper limb joints (e.g., elbow, shoulder) or distinct zonal architectures within the same diarthrodial joint may alter the vulnerability to disuse atrophy. Additionally, the mechanobiological role of chondrocytes under physiological loading has been overlooked; the absence of cyclic compression disrupts chondrocyte metabolic activity, alters gene expression profiles, and impairs the homeostatic feedback loops that maintain the pericellular matrix and hyaline cartilage integrity [67,183]. Moreover, the canine knee model proposed by Grumbles et al. [184] attributed cartilage atrophy not only to reduced chondrocyte protein synthesis but also to elevated protease activity and decreased TIMP expression, highlighting a biochemical cascade that may contribute to disuse-induced degeneration. Crucially, this cross-sectional design prevents temporal analysis of how nutritional deficits and mechanotransductive deprivation interact over time to drive overt OA. Future studies should integrate longitudinal imaging, biomechanical loading models, and molecular profiling to unravel the combined effects of nutrition and mechanical stimulation on cartilage degeneration.

The heterogeneity of the immobilization modalities presents a significant limitation. Spinal cord injury eliminates both static and dynamic loading, driving the rapid degeneration of the calcified and superficial cartilage zones, whereas rigid external fixation or cast immobilization may preserve partial joint congruence and yield divergent atrophic patterns. Joint-specific responses also vary, with hip and knee cartilage being the most susceptible to thickness loss, and smaller joints exhibit early biochemical changes. Clinical experience in athletic cohorts has demonstrated that cartilage repair techniques—ranging from microfracture to autologous chondrocyte implantation and emerging stem cell–mediated gene therapies—can restore surface integrity and facilitate return to sport, yet the optimal rehabilitation protocols to safeguard against long-term chondropenia remain undefined [182,185,186]. Concurrently, emerging diagnostic modalities, including quantitative MRI (T2 mapping, dGEMRIC), optical coherence tomography, metabolic imaging, ultrasound, mechano-acoustic sensors, and synovial fluid biomarkers such as COMP and CTX-II, offer unprecedented sensitivity for early cartilage changes but have not been systematically applied in longitudinal studies of disuse-induced degeneration [9,164,165,166,167]. Similarly, sodium MRI combined with proton MRI shows potential for the noninvasive assessment of glycosaminoglycan content in joint cartilage, yet the establishment of precise sodium concentration thresholds for early knee/hip OA detection remains a significant challenge [167]. To bridge these gaps, future research must stratify cohorts by immobilization type/mechanism and joint site, harmonize post-repair loading regimens based on the repair technique, and integrate multimodal imaging with molecular biomarkers to map the trajectory or continuum from inactivity-induced chondropenia to clinical OA.

Although the conventional ‘wear and tear’ perspective has long underscored the cumulative impact of biomechanical stress on hyaline cartilage health and has driven strategies aimed at minimizing joint overload to forestall post-traumatic OA, it nevertheless falls short in accounting for several key epidemiological and comparative observations. For instance, the inconsistent associations between high-impact activities—such as running—and incident OA [46,47] as well as other weight-bearing sports [48] challenge the notion that repetitive loading alone dictates disease onset. Paradoxically, sedentary cohorts exhibit a disproportionately high OA burden [22,29], and arboreal primates—subjected to constant joint loading—remain largely OA-free [49,50]. As discussed earlier, the classic model cannot explain the striking disparity in OA prevalence among different joints—most notably the ankle, knee, and hip [42,43]—nor does it address well the sex-specific patterns observed, whereby women experience significantly higher rates of knee and hip OA than men, despite similar cartilage thickness and generally lower PALs [44,45]. Taken together, these inconsistencies highlight the limitations of a purely degradative framework and lend credence to the emerging “use it or lose it” paradigm in avascular cartilage biology for understanding susceptibility to idiopathic OA pathogenesis in sedentary contexts.

Nonetheless, the ‘use it or lose it’ paradigm similarly encounters unresolved complexities when applied to joint-specific cartilage morphology and degeneration. The hyaline cartilage thickness across knee compartments reveals that the lateral tibiofemoral region—where cartilage is markedly thicker on both femoral and tibial surfaces—exhibits a lower incidence of OA despite being subjected to multifaceted loading regimes, whereas the medial compartment, with comparatively thinner cartilage, demonstrates the highest rates of joint space narrowing and OA. This dichotomy suggests that beyond a critical cartilage depth threshold, limitations in nutrient diffusion may cede primacy to local biomechanical factors, namely, articular incongruity and focal stress concentration. In anatomically incongruent regions, increased cartilage thickness likely represents an adaptive mechanism that appears to function as a “biomechanical buffer”, deforming under compressive load to expand the contact area and thereby redistribute focal stresses away from the subchondral bone [125]. Such biomechanical specialization underscores that both insufficient loading and excessive focal stress interact in a nuanced balance that governs the cartilage homeostasis and vulnerability to degeneration. Collectively, these findings highlight the necessity of an integrated mechanobiological framework—one that balances and synergizes anabolic stimulus (‘use it or lose it’) with protection against high-magnitude stress concentrations (‘wear and tear’)—to accurately predict site-specific susceptibility to osteoarthritic degeneration.

Building on the integrated mechanobiological paradigm described above, a novel dimension emerges when considering the paradoxical and enigmatic relationship between cigarette smoking and OA. For decades, epidemiological studies have reported conflicting associations, with some observing a reduced prevalence of knee and hip OA among smokers [187], while others have demonstrated a clear link between smoking and increased OA incidence and severity, particularly in the ankle [188]. This inconsistent and controversial relationship underscores a critical gap in our understanding of the joint-specific mechanisms by which smoking influences OA risk. To address these discrepancies, I propose the limiting-depth hypothesis, which posits that joint-specific differences in cartilage thickness across load-bearing joints govern the penetration depth of low-molecular-weight tobacco smoke solutes (e.g., nicotine and carbon monoxide) in the cartilage matrix under physiological mechanical loading and synovial fluid transport. In thicker cartilage joints, such as the knee and hip, solute diffusion remains largely superficial—rendering these articulations “passive smokers”—whereas the thinner cartilage of the ankle, under this model, allows deeper, more uniform solute infiltration, effectively making it an “active smoker” joint. This hypothesis integrates cartilage transport physiology with anatomical and biomechanical principles to explain the divergent smoking–OA associations across different diarthrodial joints [187,188]. Future research should focus on validating this hypothesis through experimental cartilage transport models, dynamic loading studies, and joint-specific biomarker profiling to better understand how the interplay between smoking, the mechanical environment, and cartilage architecture collectively shapes OA risk.

Central to the limiting-depth concept is the role of the unstirred layer at the cartilage–synovial fluid interface, a complex microenvironment that governs solute transport into the cartilage matrix. The reaction-diffusion models of cartilage have traditionally idealized the unstirred layer as a uniform, one-dimensional film with constant pH, ionic strength, and charge density, yet in vivo the unstirred layer overlays a structurally anisotropic, topographically heterogeneous surface where variable thickness, tortuous diffusion pathways, and convective microflows generate microdomains of distinct chemical milieu. Consequently, static assumptions fail to capture local acidification from lactate/proton accumulation, pH-driven shifts in nutrient solubility and ionization, dynamic Donnan equilibria, and hypoxia-induced redox gradients, all of which accelerate the degradation of acid-sensitive substrates, concentrate advanced glycation end-products (AGEs), stiffen and microcrack the superficial matrix, and alter the redox potential and antioxidant stability. Hypothetically speaking, surface-bound and fluid-phase solutes can also pre-process critical substrates (e.g., glucose, glucosamine, vitamin D precursors, pharmaceuticals), modifying their bioavailability before deeper penetration, while chondrocyte and synoviocyte feedback—through MMP upregulation and cytokine release—dynamically remodels both matrix composition and synovial fluid chemistry. Although theoretical predictions of these coupled reaction–diffusion–feedback networks are compelling, they remain largely unvalidated by direct in situ measurements of pH, O_2_ tension, and solute concentrations within living-joint unstirred layers due to substantial technical challenges. To overcome these limitations, future efforts must integrate multi-dimensional geometries, convective transport, and evolving boundary conditions with comprehensive reaction–diffusion kinetics (encompassing glucose metabolism, lactate/proton production, AGE and redox chemistries, antioxidant degradation, lipid oxidation, and toxin metabolism) and cell–matrix signaling into computational frameworks rigorously calibrated against high-resolution experimental data. Interdisciplinary collaborations—leveraging microelectrode arrays, molecular probes, and advanced imaging—will thus redefine the unstirred layer as an active “microreactor” rather than a passive diffusion barrier, enabling precise solute-flux prediction, identification of novel therapeutic targets, and development of strategies that both attenuate chemically mediated superficial matrix breakdown and overcome diffusional constraints to prevent subsurface cartilage degeneration, thereby safeguarding joint integrity in aging and disease.

## 15. Concluding Remarks and Recommendations

This study highlights the potential impact of cartilage thickness and prolonged sedentary behavior on the early development of OA, introducing the term *sedenthrosis*—or alternatively, *sedenthritis*—to describe this synergistic and emerging association. Through the lens of the ‘sedentary–thickness axis’ framework—which incorporates often-overlooked factors such as cartilage thickness and the persistence of synovial fluid films—it is proposed that a reduced limiting diffusion depth may contribute to early pathological changes in the deeper zones of cartilage. These alterations may define a unique molecular endotype of knee and/or hip OA in sedentary individuals—one that standard radiographic techniques often miss because it involves subtle metabolic shifts and reductions in highly sulfated glycosaminoglycans, particularly those linked to aggrecan [167].

The principles described by simplified Equations (1) and (2) succinctly but aptly reveal a critical and often underestimated dual challenge in cartilage physiology: excessive cartilage thickness not only impairs nutrient diffusion but also hinders the removal of catabolic byproducts, promoting their accumulation in the deep cartilage strata and precipitating ECM degradation [31,32,150]. Such a “metabolic bottleneck” not only accelerates matrix degradation but also contributes to chondrocyte death, driving OA progression. This limitation poses a significant risk to cartilage health, potentially accelerating joint degeneration, particularly in sedentary lifestyles, where reduced physical activity/exercise exacerbates nutrient deprivation caused by stagnant fluid layers. Addressing this challenge is essential for preserving cartilage integrity and mitigating the risk of OA development.

Intriguingly, diffusion-resistant boundary layers on all diarthrodial (movable) synovial joint surfaces introduce an additional (quintessential) diffusion barrier that becomes particularly significant in articular cartilage exceeding 1 mm in thickness, thereby slowing solute exchange and potentially disrupting chondrocyte metabolism within the deep tissue [32,37]. These findings emphasize the urgent need for further targeted research on these metabolic mechanisms to better understand OA pathogenesis. This study also underscores the critical role of regular physical activity/exercise for maintaining joint health and preventing cartilage degradation. Considering the widespread sedentary behavior in developed countries, it is imperative that healthcare providers and policymakers prioritize the promotion of active lifestyles to mitigate the risk of OA and its associated comorbidities [175].

Yet, to effectively address the complexities of early-stage OA, it is essential to conceptualize cartilage degeneration in terms of zonal involvement, distinguishing between “superficial” and “deep” patterns, or categories, based on their anatomical proximity to the tangential and radial zones, respectively. This classification can enhance mechanistic insights into the distinct etiopathophysiological processes governing each zone, deepen our understanding of disease progression, and guide the development of precise diagnostic and therapeutic approaches. Embracing a zonal paradigm may improve early detection by capturing spatially localized degeneration and support the design of mechanistically informed interventions—including tailored biomechanical protocols and nutrient delivery approaches—aimed at preserving deep cartilage viability and function. However, this field remains far from mature.

Future research should focus on elucidating the underlying mechanisms of OA pathogenesis in the context of disuse atrophy (chondropenia) using advanced computational approaches—including depth-resolved reaction-diffusion finite element analyses and agent-based metabolic simulations—to capture spatial heterogeneity, transient transport dynamics, and mechanobiological feedback within joint cartilage. In parallel, the concept of *sedenthrosis* warrants validation in longitudinal clinical cohorts using noninvasive biomarkers of cartilage metabolism to establish its clinical relevance, characterize its phenotype-specific pathophysiological distinctiveness, and define its role within early OA subtypes [59,60,61]. Amid contemporary sedentary habits, rethinking OA from a ‘wear and tear’ perspective to a ‘move it or lose it’ approach underscores the preventive power and therapeutic value of physical activity/exercise, with the potential not only to reduce the burden of knee and hip OA but also to enhance quality of life by driving more effective strategies [66,70]. In essence, movement may be the indispensable medicine for thick cartilage—a critical chondroprotective “dose” that should not be overlooked.

To effectively tackle the metabolic imbalances resulting from nutrient deprivation in deep cartilage, especially among sedentary individuals, a comprehensive and integrated approach is not merely beneficial—it is imperative. As we navigate the intricate relationship between cartilage health and the increasingly inactive lifestyles of modern society, we must advocate pioneering and innovative strategies to ensure optimal nutrient delivery and encourage physical activity/exercise. Robust international collaboration among biomechanics, computational modeling, and clinical science is crucial to close critical knowledge gaps in cartilage health and disease, enabling in-depth characterization of thickness-dependent differences in hyaline cartilage and driving the development of precision strategies for the prevention and management of OA in an increasingly sedentary population. Taken together, these interdisciplinary and collaborative efforts will pave the way for next-generation strategies for OA prevention and management, ultimately leading to improved clinical outcomes. Without such efforts, the insidious, gradual, and often undetected degeneration of thick articular cartilage in sedentary cohorts risks becoming a major public health concern.

## Figures and Tables

**Table 1:** Structural and functional characteristics of hyaline cartilage zones.

Zone	Description	Function
Superficial (Tangential)	Thinnest layer; flattened chondrocytes oriented parallel to surface; densely packed, horizontally aligned collagen fibers; lowest proteoglycan content, highest water content	Provides a smooth, low-friction surface; resists shear forces; maintains lubrication; protects deeper layers from tensile stress
Middle (Transitional)	Intermediate thickness; spherical chondrocytes arranged randomly; collagen fibers oriented obliquely in a Benninghoff arcade-like pattern; moderate proteoglycan content and water content	Acts as a buffer between superficial and deep zones; absorbs compressive forces; aids in load distribution; resists tensile and compressive stresses
Deep (Radial)	Thickest layer; large, columnar chondrocytes aligned perpendicular to surface; highest proteoglycan content; lower water content; vertically oriented collagen fibers	Provides the greatest resistance to compressive forces; supports load-bearing function; anchors cartilage to the underlying calcified zone and subchondral bone for structural integrity
Tidemark	Thin, distinct boundary between non-calcified cartilage and calcified cartilage; visible in histological sections; undergoes thickening in OA	Denotes the transition to calcified cartilage; critical in calcification processes and cartilage integrity.
Calcified cartilage	Thin zone adjacent to subchondral bone; small, hypertrophic chondrocytes embedded in a mineralized matrix; hydroxyapatite crystals present; lowest water and proteoglycan content	Anchors cartilage to subchondral bone; provides mechanical stability by integrating cartilage with bone; critical for transferring loads from soft cartilage to rigid bone; involved in cartilage repair and remodeling

**Table 2:** Integrated physical, compositional and functional properties of articular cartilage surface–associated layers.

Layer	Thickness (µm)	Main Components	Function
Unstirred synovial layer	~50–>1000 (flow/geometry-dependent)	Water (~95% WW), hyaluronic acid (3–4 mg/mL), PRG4/lubricin (~200 µg/mL), DPPC (0.1–0.2 mg/mL), albumin (~12 mg/mL), γ-globulins (~7 mg/mL), electrolytes (Na^+^, Cl^−^, K^+^, Ca^2+^), O_2_, CO_2_, glucose, lactate, other metabolites	Regulates solute exchange and effective permeability; flow-controlled diffusion; restricts passive transport during low-flow conditions
Lamina splendens	3–8	Dense amorphous surface coat (tight mesh), acellular; densely packed collagen fibrils (random orientation), hyaluronan, PRG4, fibronectin, surface-active phospholipids (SAPL)	Boundary lubrication; ultra-low friction coefficient (COF ~ 0.005), often referred to as superlubricity; solute exchange interface; surface sealing and low-permeability barrier
Superficial tangential zone	100–200	Type II and IX collagen (parallel), aggrecan (2–4% WW), PRG4, minor elastin, interstitial water (~85% WW)	Primary load-bearing zone; maintains tensile strength and surface integrity; regulates mechanochemical signaling

## Data Availability

No new data were created or analyzed in this study. Data sharing is not applicable to this article.

## Abbreviations

The following abbreviations are used in this manuscript:

ADAMTS	A Disintegrin and Metalloproteinase with Thrombospondin Motifs
ADLs	Activities of Daily Living
AGEs	Advanced Glycation End-products
ATP	Adenosine Triphosphate
COF	Coefficient of Friction
COMP	Cartilage Oligomeric Matrix Protein
dGEMRIC	Delayed Gadolinium-Enhanced Magnetic Resonance Imaging of Cartilage
DPPC	Dipalmitoylphosphatidylcholine
ECM	Extracellular Matrix
FCD	Fixed Charge Density
(GLA:D)	Good Life with osteoArthritis in Denmark
Glc	Glucose
GLUT	Glucose Transporter
IL-1β	Interleukin-1 beta
MMP	Matrix Metalloproteinase
MRI	Magnetic Resonance Imaging
OA	Osteoarthritis
PALs	Physical Activity Levels
PET	Positron Emission Tomography
PRG4	Proteoglycan 4
SAPL	Surface-Active Phospholipid
STZ	Superficial Tangential Zone
TIMP	Tissue Inhibitor of Metalloproteinases
TNF-α	Tumor Necrosis Factor-alpha
WHO	World Health Organization
WW	Wet Weight

## References

[1] Steinmetz J.D., Culbreth G.T., Haile L.M., Rafferty Q., Lo J., Fukutaki K.G., Cruz J.A., Smith A.E., Vollset S.E., Brooks P.M. (2023). Global, regional, and national burden of osteoarthritis, 1990-2020 and projections to 2050: A systematic analysis for the Global Burden of Disease Study 2021. Lancet Rheumatol..

[2] Maradit Kremers H., Larson D.R., Crowson C.S., Kremers W.K., Washington R.E., Steiner C.A., Jiranek W.A., Berry D.J. (2015). Prevalence of total hip and knee replacement in the United States. J. Bone Jt. Surg. Am..

[3] Fernandes L., Hagen K.B., Bijlsma J.W., Andreassen O., Christensen P., Conaghan P.G., Doherty M., Geenen R., Hammond A., Kjeken I. (2013). EULAR recommendations for the non-pharmacological core management of hip and knee osteoarthritis. Ann. Rheum. Dis..

[4] Musumeci G., Aiello F.C., Szychlinska M.A., Di Rosa M., Castrogiovanni P., Mobasheri A. (2015). Osteoarthritis in the XXIst century: Risk factors and behaviours that influence disease onset and progression. Int. J. Mol. Sci..

[5] Berenbaum F., Wallace I.J., Lieberman D.E., Felson D.T. (2018). Modern-day environmental factors in the pathogenesis of osteoarthritis. Nat. Rev. Rheumatol..

[6] Culvenor A.G., Øiestad B.E., Hart H.F., Stefanik J.J., Guermazi A., Crossley K.M. (2019). Prevalence of knee osteoarthritis features on magnetic resonance imaging in asymptomatic uninjured adults: A systematic review and meta-analysis. Br. J. Sports Med..

[7] Losina E., Thornhill T.S., Rome B.N., Wright J., Katz J.N. (2012). The dramatic increase in total knee replacement utilization rates in the United States cannot be fully explained by growth in population size and the obesity epidemic. J. Bone Jt. Surg. Am..

[8] Shichman I., Roof M., Askew N., Nherera L., Rozell J.C., Seyler T.M., Schwarzkopf R. (2023). Projections and epidemiology of primary hip and knee arthroplasty in medicare patients to 2040–2060. JB JS Open Access.

[9] Mobasheri A., van Spil W.E., Budd E., Uzieliene I., Bernotiene E., Bay-Jensen A.C., Larkin J., Levesque M.C., Gualillo O., Henrotin Y. (2019). Molecular taxonomy of osteoarthritis for patient stratification, disease management and drug development: Biochemical markers associated with emerging clinical phenotypes and molecular endotypes. Curr. Opin. Rheumatol..

[10] Man G.S., Mologhianu G. (2014). Osteoarthritis pathogenesis—A complex process that involves the entire joint *J*. Med. Life.

[11] Kroon F.P.B., Veenbrink A.I., de Mutsert R., Visser A.W., van Dijk K.W., le Cessie S., Rosendaal F.R., Kloppenburg M. (2019). The role of leptin and adiponectin as mediators in the relationship between adiposity and hand and knee osteoarthritis. Osteoarthr. Cartil..

[12] Andriacchi T.P., Griffin T.M., Loeser R.F., Chu C.R., Roos E.M., Hawker G.A., Erhart-Hledik J.C., Fischer A.G. (2020). Bridging Disciplines as a pathway to Finding New Solutions for Osteoarthritis a collaborative program presented at the 2019 Orthopaedic Research Society and the Osteoarthritis Research Society International. Osteoarthr. Cartil. Open.

[13] Krakowski P., Rejniak A., Sobczyk J., Karpinski R. (2024). Cartilage integrity: A review of mechanical and frictional properties and repair approaches in osteoarthritis. Healthcare.

[14] Lai W.M., Hou J.S., Mow V.C. (1991). A triphasic theory for the swelling and deformation behaviors of articular cartilage. J. Biomech. Eng..

[15] Radin E.L., Burr D.B., Caterson B., Fyhrie D., Brown T.D., Boyd R.D. (1991). Mechanical determinants of osteoarthrosis. Semin. Arthritis Rheum..

[16] Thambyah A. (2005). A hypothesis matrix for studying biomechanical factors associated with the initiation and progression of posttraumatic osteoarthritis. Med. Hypotheses.

[17] Andriacchi T.P., Mundermann A. (2006). The role of ambulatory mechanics in the initiation and progression of knee osteoarthritis. Curr. Opin. Rheumatol..

[18] Hosseininia S., Lindberg L.R., Dahlberg L.E. (2013). Cartilage collagen damage in hip osteoarthritis similar to that seen in knee osteoarthritis; a case-control study of relationship between collagen, glycosaminoglycan and cartilage swelling. BMC Musculoskelet. Disord..

[19] Petersson I.F. (1996). Occurrence of osteoarthritis of the peripheral joints in European populations. Ann. Rheum. Dis..

[20] Inoue K., Hukuda S., Fardellon P., Yang Z.Q., Nakai M., Katayama K., Ushiyama T., Saruhashi Y., Huang J., Mayeda A. (2001). Prevalence of large-joint osteoarthritis in Asian and Caucasian skeletal populations. Rheumatology.

[21] Wallace I.J., Worthington S., Felson D.T., Jurmain R.D., Wren K.T., Maijanen H., Woods R.J., Lieberman D.E. (2017). Knee osteoarthritis has doubled in prevalence since the mid-20th century. Proc. Natl. Acad. Sci. USA.

[22] Safiri S., Kolahi A.-A., Smith E., Hill C., Bettampadi D., Mansournia M.A., Hoy D., Ashrafi-Asgarabad A., Sepidarkish M., Almasi-Hashiani A. (2020). Global, regional and national burden of osteoarthritis 1990–2017: A systematic analysis of the Global Burden of Disease Study 2017. Ann. Rheum. Dis..

[23] Katz J.N., Arant K.R., Loeser R.F. (2021). Diagnosis and treatment of hip and knee osteoarthritis: A review. JAMA.

[24] Bramble D.M., Lieberman D.E. (2004). Endurance running and the evolution of Homo. Nature.

[25] Pontzer H. (2017). Economy and endurance in human evolution. Curr. Biol..

[26] Carter D.R., Caler W.E. (1985). A cumulative damage model for bone fracture. J. Orthop. Res..

[27] Frost H.M. (1999). Joint anatomy, design, and arthroses: Insights of the Utah paradigm. Anat. Rec..

[28] Szczodry M., Coyle C.H., Kramer S.J., Smolinski P., Chu C.R. (2009). Progressive chondrocyte death after impact injury indicates a need for chondroprotective therapy. Am. J. Sports Med..

[29] Guthold R., Stevens G.A., Riley L.M., Bull F.C. (2018). Worldwide trends in insufficient physical activity from 2001 to 2016: A pooled analysis of 358 population-based surveys with 1.9 million participants. Lancet Glob. Health.

[30] Wallace I.J., Bendele A.M., Riew G., Frank E.H., Hung H.H., Holowka N.B., Bolze A.S., Venable E.M., Yegian A.K., Dingwall H.L. (2019). Physical inactivity and knee osteoarthritis in guinea pigs. Osteoarthr. Cartil..

[31] Maroudas A., Bullough P., Swanson S.A.V., Freeman M.A.R. (1968). The permeability of articular cartilage. J. Bone Jt. Surg..

[32] Stockwell R.A. (1979). Biology of Cartilage Cells.

[33] O'Hara B.P., Urban J.P., Maroudas A. (1990). Influence of cyclic loading on the nutrition of articular cartilage. Ann. Rheum. Dis..

[34] Levick J.R. (1995). Microvascular architecture and exchange in synovial joints. Microcirculation.

[35] Zhou S., Cui Z., Urban J.P.G. (2004). Factors influencing the oxygen concentration gradient from the synovial surface of articular cartilage to the cartilage-bone interface: A modeling study. Arthritis Rheum..

[36] Torzilli P.A. (1993). Effects of temperature, concentration and articular surface removal on transient solute diffusion in articular cartilage. Med. Biol. Eng. Comput..

[37] Wang Y., Wei L., Zeng L., He D., Wei X. (2013). Nutrition and degeneration of articular cartilage. Knee Surg. Sports Traumatol. Arthrosc..

[38] Moskowitz R.W., Howell D., Goldberg V., Mankin H. (2007). Osteoarthritis: Diagnosis and Medical/Surgical Management.

[39] Abramson S.B., Attur M. (2009). Developments in the scientific understanding of osteoarthritis. Arthritis Res. Ther..

[40] Radin E., Paul I., Rose R. (1972). Role of mechanical factors in pathogenesis of primary osteoarthritis. Lancet.

[41] Gardner D.L. (1983). The nature and causes of osteoarthrosis. Br. Med. J..

[42] Cushnaghan J., Dieppe P. (1991). Study of 500 patients with limb joint osteoarthritis. I. Analysis by age, sex, and distribution of symptomatic joint sites. Ann. Rheum. Dis..

[43] Saltzman C.L., Salamon M.L., Blanchard G.M., Huff T., Hayes A., Buckwalter J.A., Amendola A. (2005). Epidemiology of ankle arthritis. Report of a consecutive series of 639 patients from a tertiary orthopaedic center. Iowa Orthop. J..

[44] Srikanth V.K., Fryer J.L., Zhai G., Winzenberg T.M., Hosmer D., Jones G. (2005). A meta-analysis of sex differences prevalence, incidence and severity of osteoarthritis. Osteoarthr. Cartil..

[45] O'Connor M.I. (2007). Sex differences in osteoarthritis of the hip and knee. J. Am. Acad. Orthop. Surg..

[46] Ponzio D.Y., Syed U.A.M., Purcell K., Cooper A.M., Maltenfort M., Shaner J., Chen A.F. (2018). Low prevalence of hip and knee arthritis in active marathon runners. J. Bone Jt. Surg. Am..

[47] Hartwell M.J., Tanenbaum J.E., Chiampas G., Terry M.A., Tjong V.K. (2023). Does running increase the risk of hip and knee arthritis? A survey of 3804 marathon runners. Sports Health.

[48] Babayeva N., Donmez G., Ozcakar L., Torgutalp S.S., Karacoban L., Gedik E., Korkusuz F., Doral M.N. (2021). Mean femoral cartilage thickness is higher in athletes as compared with sedentary individuals. Knee Surg. Sports Traumatol. Arthrosc..

[49] Rothschild B.M., Woods R.J. (1993). Arthritis in new world monkeys: Osteoarthritis, calcium pyrophosphate deposition disease, and spondyloarthropathy. Int. J. Primatol..

[50] Jurmain R. (2000). Degenerative joint disease in African great apes: An evolutionary perspective. J. Hum. Evol..

[51] Buckwalter J.A. (1995). Osteoarthritis and articular cartilage use, disuse, and abuse: Experimental studies. J. Rheumatol. Suppl..

[52] Gregory M.H., Capito N., Kuroki K., Stoker A.M., Cook J.L., Sherman S.L. (2012). A review of translational animal models for knee osteoarthritis. Arthritis.

[53] Kuyinu E.L., Narayanan G., Nair L.S., Laurencin C.T. (2016). Animal models of osteoarthritis: Classification, update, and measurement of outcomes. J. Orthop. Surg. Res..

[54] Takahashi I., Matsuzaki T., Kuroki H., Hoso M. (2021). Disuse atrophy of articular cartilage induced by unloading condition accelerates histological progression of osteoarthritis in a post-traumatic rat model. Cartilage.

[55] Rizzi L., Turati M., Bresciani E., Anghilieri F.M., Meanti R., Molteni L., Piatti M., Zanchi N., Coco S., Buonanotte F. (2022). Characterization of microRNA levels in synovial fluid from knee osteoarthritis and anterior cruciate ligament tears. Biomedicines.

[56] Xu L., Kazezian Z., Pitsillides A.A., Bull A.M.J. (2024). A synoptic literature review of animal models for investigating the biomechanics of knee osteoarthritis. Front. Bioeng. Biotechnol..

[57] Felson D.T. (2013). Osteoarthritis as a disease of mechanics. Osteoarthr. Cartil..

[58] Martin J.A., Buckwalter J.A. (2002). Aging, articular cartilage chondrocyte senescence and osteoarthritis. Biogerontology.

[59] Mobasheri A., Saarakkala S., Finnila M., Karsdal M.A., Bay-Jensen A.C., van Spil W.E. (2019). Recent advances in understanding the phenotypes of osteoarthritis. F1000Research.

[60] Deveza L.A., Nelson A.E., Loeser R.F. (2019). Phenotypes of osteoarthritis: Current state and future implications. Clin. Exp. Rheumatol..

[61] Hart D.A. (2022). Osteoarthritis as an umbrella term for different subsets of humans undergoing joint degeneration: The need to address the differences to develop effective conservative treatments and prevention strategies. Int. J. Mol. Sci..

[62] Vanwanseele B., Eckstein F., Knecht H., Spaepen A., Stussi E. (2003). Longitudinal analysis of cartilage atrophy in the knees of patients with spinal cord injury. Arthritis Rheum..

[63] Hinterwimmer S., Krammer M., Krötz M., Glaser C., Baumgart R., Reiser M., Eckstein F. (2004). Cartilage atrophy in the knees of patients after seven weeks of partial load bearing. Arthritis Rheum..

[64] Nomura M., Sakitani N., Iwasawa H., Kohara Y., Takano S., Wakimoto Y., Kuroki H., Moriyama H. (2017). Thinning of articular cartilage after joint unloading or immobilization. An experimental investigation of the pathogenesis in mice. Osteoarthr. Cartil..

[65] Liphardt A.M., Mündermann A., Andriacchi T.P., Achtzehn S., Heer M., Mester J. (2018). Sensitivity of serum concentration of cartilage biomarkers to 21-days of bed rest. J. Orthop. Res..

[66] Castrogiovanni P., Di Rosa M., Ravalli S., Castorina A., Guglielmino C., Imbesi R., Vecchio M., Drago F., Szychlinska M.A., Musumeci G. (2019). Moderate physical activity as a prevention method for knee osteoarthritis and the role of synoviocytes as biological key. Int. J. Mol. Sci..

[67] Vincent T.L., Wann A.K.T. (2019). Mechanoadaptation: Articular cartilage through thick and thin. J. Physiol..

[68] Ganse B., Cucchiarini M., Madry H. (2022). Joint cartilage in long-duration spaceflight. Biomedicines.

[69] del Río E. (2024). A novel etiological approach for the development of knee osteoarthritis in sedentary adults. Med. Hypotheses.

[70] Nissen N., Holm P.M., Bricca A., Dideriksen M., Tang L.H., Skou S.T. (2022). Clinicians’ beliefs and attitudes to physical activity and exercise therapy as treatment for knee and/or hip osteoarthritis: A scoping review. Osteoarthr. Cartil..

[71] Bull F.C., Al-Ansari S.S., Biddle S., Borodulin K., Buman M.P., Cardon G., Carty C., Chaput J.P., Chastin S., Chou R. (2020). World Health Organization 2020 guidelines on physical activity and sedentary behaviour. Br. J. Sports Med..

[72] Lieberman D.E., Kistner T.M., Richard D., Lee I.M., Baggish A.L. (2021). The active grandparent hypothesis: Physical activity and the evolution of extended human healthspans and lifespans. Proc. Natl. Acad. Sci. USA.

[73] World Health Organization (2020). Registered Vehicles. Data by Country. https://apps.who.int/gho/data/node.main.A995.

[74] Wikipedia List of Countries and Territories by Motor Vehicles per Capita. https://en.wikipedia.org/wiki/List_of_countries_and_territories_by_motor_vehicles_per_capita#cite_ref-3.

[75] McKenzie B. (2014). Modes Less Traveled—Bicycling and Walking to Work in the United States: 2008–2012.

[76] Barbour K.E., Helmick C.G., Boring M., Zhang X., Lu H., Holt J.B. (2016). Prevalence of doctor-diagnosed arthritis at state and county levels—United States, 2014. MMWR Morb. Mortal. Wkly. Rep..

[77] An R., Xiang X., Yang Y., Yan H. (2016). Mapping the prevalence of physical inactivity in U.S. states, 1984–2015. PLoS ONE.

[78] Carter D.R., Beaupre G.S., Wong M., Smith R.L., Andriacchi T.P., Schurman D.J. (2004). The mechanobiology of articular cartilage development and degeneration. Clin. Orthop. Relat. Res..

[79] Mackie E.J., Ahmed Y.A., Tatarczuch L., Chen K.S., Mirams M. (2008). Endochondral ossification: How cartilage is converted into bone in the developing skeleton. Int. J. Biochem. Cell Biol..

[80] Sharma A., Jagga S., Lee S.-S., Nam J.-S. (2013). Interplay between cartilage and subchondral bone contributing to pathogenesis of osteoarthritis. Int. J. Mol. Sci..

[81] Salazar V.S., Gamer L.W., Rosen V. (2016). BMP signalling in skeletal development, disease and repair. Nat. Rev. Endocrinol..

[82] Xiao Z.-f., Su G.-y., Hou Y., Chen S.-d., Lin D.-k. (2018). Cartilage degradation in osteoarthritis: A process of osteochondral remodeling resembles the endochondral ossification in growth plate?. Med. Hypotheses.

[83] Li Y., Liem Y., Zamli Z., Sullivan N., Dall'Ara E., Ahmed H., Sellers G.M., Blom A., Sharif M. (2021). Subchondral bone microarchitectural and mineral properties and expression of key degradative proteinases by chondrocytes in human hip osteoarthritis. Biomedicines.

[84] Yokota S., Ishizu H., Miyazaki T., Takahashi D., Iwasaki N., Shimizu T. (2024). Osteoporosis, osteoarthritis, and subchondral insufficiency fracture: Recent insights. Biomedicines.

[85] Valderrabano V., Horisberger M., Russell I., Dougall H., Hintermann B. (2009). Etiology of ankle osteoarthritis. Clin. Orthop. Relat. Res..

[86] Horisberger M., Valderrabano V., Hintermann B. (2009). Posttraumatic ankle osteoarthritis after ankle-related fractures. J. Orthop. Trauma.

[87] Herrera-Pérez M., González-Martín D., Vallejo-Márquez M., Godoy-Santos A.L., Valderrabano V., Tejero S. (2021). Ankle osteoarthritis aetiology. J. Clin. Med..

[88] Brown T.D., Johnston R.C., Saltzman C.L., Marsh J.L., Buckwalter J.A. (2006). Posttraumatic osteoarthritis: A first estimate of incidence, prevalence, and burden of disease. J. Orthop. Trauma.

[89] Michael J.W.P., Schluter-Brust K.U., Eysel P. (2010). The epidemiology, etiology, diagnosis, and treatment of osteoarthritis of the knee. Dtsch. Arztebl. Int..

[90] Eckstein F., Tieschky M., Faber S., Englmeier K.H., Reiser M. (1999). Functional analysis of articular cartilage deformation, recovery, and fluid flow following dynamic exercise in vivo. Anat. Embryol..

[91] Brand R.A. (2005). Joint contact stress: A reasonable surrogate for biological processes?. IOWA Orthop. J..

[92] Fukui T., Ueda Y., Kamijo F. (2016). Ankle, knee, and hip joint contribution to body support during gait. J. Phys. Ther. Sci..

[93] Sanford B., Williams J., Zucker-Levin A., Mihalko W. (2014). Hip, knee, and ankle joint forces in healthy weight, overweight, and obese individuals during walking. Computational Biomechanics for Medicine: Fundamental Science and Patient-Specific Applications.

[94] Eberhardt A.W., Keer L.M., Lewis J.L., Vithoontien V. (1990). An analytical model of joint contact. J. Biomech. Eng..

[95] Ihn J.C., Kim S.J., Park I.H. (1993). In vitro study of contact area and pressure distribution in the human knee after partial and total meniscectomy. Int. Orthop..

[96] Brown T.D., Shaw D.T. (1983). In vitro contact stress distributions in the natural human hip. J. Biomech..

[97] Kimizuka M., Kurosawa H., Fukubayashi T. (1980). Load-bearing pattern of the ankle joint. Contact area and pressure distribution. Arch. Orthop. Trauma Surg..

[98] Kempson G.E. (1991). Age-related changes in the tensile properties of human articular cartilage: A comparative study between the femoral head of the hip joint and the talus of the ankle joint. Biochim. Biophys. Acta.

[99] Nelson F., Dahlberg L., Laverty S., Reiner A., Pidoux I., Ionescu M., Fraser G.L., Brooks E., Tanzer M., Rosenberg L.C. (1998). Evidence for altered synthesis of type II collagen in patients with osteoarthritis. J. Clin. Investig..

[100] Lephart S.M., Pincivero D.M., Rozzi S.L. (1998). Proprioception of the ankle and knee. Sports Med..

[101] Treppo S., Koepp H., Quan E.C., Cole A.A., Kuettner K.E., Grodzinsky A.J. (2000). Comparison of biomechanical and biochemical properties of cartilage from human knee and ankle pairs. J. Orthop. Res..

[102] Schumacher B.L., Su J.-L., Lindley K.M., Kuettner K.E., Cole A.A. (2002). Horizontally oriented clusters of multiple chondrons in the superficial zone of ankle, but not knee articular cartilage. Anat. Rec..

[103] Eger W., Schumacher B.L., Mollenhauer J., Kuettner K.E., Cole A.A. (2002). Human knee and ankle cartilage explants: Catabolic differences. J. Orthop. Res..

[104] Riemann B.L., Myers J.B., Lephart S.M. (2003). Comparison of the ankle, knee, hip, and trunk corrective action shown during single-leg stance on firm, foam, and multiaxial surfaces. Arch. Phys. Med. Rehabil..

[105] Kuettner K.E., Cole A.A. (2005). Cartilage degeneration in different human joints. Osteoarthr. Cartil..

[106] Hemmerich A., Brown H., Smith S., Marthandam S.S., Wyss U.P. (2006). Hip, knee, and ankle kinematics of high range of motion activities of daily living. J. Orthop. Res..

[107] Wilson W., van Burken C., van Donkelaar C., Buma P., van Rietbergen B., Huiskes R. (2006). Causes of mechanically induced collagen damage in articular cartilage. J. Orthop. Res..

[108] Catterall J.B., Hsueh M.F., Stabler T.V., McCudden C.R., Bolognesi M., Zura R., Jordan J.M., Renner J.B., Feng S., Kraus V.B. (2012). Protein modification by deamidation indicates variations in joint extracellular matrix turnover. J. Biol. Chem..

[109] den Hollander W., Ramos Y.F.M., Bos S.D., Bomer N., van der Breggen R., Lakenberg N., de Dijcker W.J., Duijnisveld B.J., Slagboom P.E., Nelissen R.G.H.H. (2014). Knee and hip articular cartilage have distinct epigenomic landscapes: Implications for future cartilage regeneration approaches. Ann. Rheum. Dis..

[110] Catterall J.B., Zura R.D., Bolognesi M.P., Kraus V.B. (2016). Aspartic acid racemization reveals a high turnover state in knee compared with hip osteoarthritic cartilage. Osteoarthr. Cartil..

[111] Knudson C.B., Knudson W. (2001). Cartilage proteoglycans. Semin. Cell Dev. Biol..

[112] Wilson W., van Donkelaar C.C., van Rietbergen B., Ito K., Huiskes R. (2004). Stresses in the local collagen network of articular cartilage: A poroviscoelastic fibril-reinforced finite element study. J. Biomech..

[113] Bhosale A.M., Richardson J.B. (2008). Articular cartilage: Structure, injuries and review of management. Br. Med. Bull..

[114] Sophia Fox A.J., Bedi A., Rodeo S.A. (2009). The basic science of articular cartilage. Structure, composition, and function. Sports Health.

[115] Eschweiler J., Horn N., Rath B., Betsch M., Baroncini A., Tingart M., Migliorini F. (2021). The biomechanics of cartilage—An overview. Life.

[116] Mow V.C., Kuei S.C., Lai W.M., Armstrong C.G. (1980). Biphasic creep and stress relaxation of articular cartilage in compression? Theory and experiments. J. Biomech. Eng..

[117] Maroudas A., Schneiderman R. (1987). "Free" and "exchangeable" or "trapped" and "non-exchangeable" water in cartilage. J. Orthop. Res..

[118] Chubinskaya S., Kuettner K.E., Cole A.A. (1999). Expression of matrix metalloproteinases in normal and damaged articular cartilage from human knee and ankle joints. Lab. Investig..

[119] Shelburne K.B., Torry M.R., Pandy M.G. (2006). Contributions of muscles, ligaments, and the ground-reaction force to tibiofemoral joint loading during normal gait. J. Orthop. Res..

[120] Eckstein F., Eisenhart-Rothe R.v., Landgraf J., Adam C., Loehe F., Müller-Gerbl M., Putz R. (1997). Quantitative analysis of incongruity, contact areas and cartilage thickness in the human hip joint. Cells Tissues Organs.

[121] Mow V.C., Ratcliffe A., Poole A.R. (1992). Cartilage and diarthrodial joints as paradigms for hierarchical materials and structures. Biomaterials.

[122] Shepherd D., Seedhom B. (1999). Thickness of human articular cartilage in joints of the lower limb. Ann. Rheum. Dis..

[123] Hogervorst T., Eilander W., Fikkers J.T., Meulenbelt I. (2012). Hip ontogenesis: How evolution, genes, and load history shape hip morphotype and cartilotype. Clin. Orthop. Relat. Res..

[124] Aurich M., Poole A.R., Reiner A., Mollenhauer C., Margulis A., Kuettner K.E., Cole A.A. (2002). Matrix homeostasis in aging normal human ankle cartilage. Arthritis Rheum..

[125] Simon W.H. (1971). Scale effects in animal joints. II. Thickness and elasticity in the deformability of articular cartilage. Arthritis Rheum..

[126] Frisbie D.D., Cross M.W., McIlwraith C.W. (2006). A comparative study of articular cartilage thickness in the stifle of animal species used in human pre-clinical studies compared to articular cartilage thickness in the human knee. Vet. Comp. Orthop. Traumatol..

[127] Malda J., de Grauw J., Benders K., Kik M., Lest C., Creemers L., Dhert W., van Weeren P. (2013). Of mice, men and elephants: The relation between articular cartilage thickness and body mass. PLoS ONE.

[128] Stockwell R.A. (1971). The interrelationship of cell density and cartilage thickness in mammalian articular cartilage. J. Anat..

[129] Huch K. (2001). Knee and ankle: Human joints with different susceptibility to osteoarthritis reveal different cartilage cellularity and matrix synthesis in vitro. Arch. Orthop. Trauma Surg..

[130] Jadin K.D., Wong B.L., Bae W.C., Li K.W., Williamson A.K., Schumacher B.L., Price J.H., Sah R.L. (2005). Depth-varying density and organization of chondrocytes in immature and mature bovine articular cartilage assessed by 3D imaging and analysis. J. Histochem. Cytochem..

[131] Otte P. (1991). Basic cell metabolism of articular cartilage. Manometric studies. Z. Rheumatol..

[132] Johnson K., Jung A., Murphy A., Andreyev A., Dykens J., Terkeltaub R. (2000). Mitochondrial oxidative phosphorylation is a downstream regulator of nitric oxide effects on chondrocyte matrix synthesis and mineralization. Arthritis Rheum..

[133] Mow V.C., Holmes M.H., Michael Lai W. (1984). Fluid transport and mechanical properties of articular cartilage: A review. J. Biomech..

[134] Hunziker E.B., Quinn T.M., Hauselmann H.J. (2002). Quantitative structural organization of normal adult human articular cartilage. Osteoarthr. Cartil..

[135] Rolauffs B., Margulis A., Kuettner K., Cole A. The cell density of the superficial layer of adult human articular cartilage is joint-specific and is altered by age and degenerative changes. Proceedings of the 48th Annual Meeting of the Orthopaedic Research Society.

[136] Johnson D.H., Pedowitz R.A. (2007). Practical Orthopaedic Sports Medicine and Arthroscopy.

[137] Teshima R., Otsuka T., Takasu N., Yamagata N., Yamamoto K. (1995). Structure of the most superficial layer of articular cartilage. J. Bone Jt. Surg. Br..

[138] Poole C.A. (1997). Articular cartilage chondrons: Form, function and failure. J. Anat..

[139] Pettenuzzo S., Arduino A., Belluzzi E., Pozzuoli A., Fontanella C.G., Ruggieri P., Salomoni V., Majorana C., Berardo A. (2023). Biomechanics of chondrocytes and chondrons in healthy conditions and osteoarthritis: A review of the mechanical characterisations at the microscale. Biomedicines.

[140] Urban J.P., Smith S., Fairbank J.C. (2004). Nutrition of the intervertebral disc. Spine.

[141] Brandt M.D., Malone J.B., Kean T.J. (2025). Advances and challenges in the pursuit of disease-modifying osteoarthritis drugs: A review of 2010-2024 clinical trials. Biomedicines.

[142] Helfferich F.G. (1962). Ion Exchange.

[143] Hunter W. (1743). Of the structure and disease of articulating cartilage. Philos. Trans. R. Soc..

[144] Mobasheri A., Vannucci S.J., Bondy C.A., Carter S.D., Innes J.F., Arteaga M.F., Trujillo E., Ferraz I., Shakibaei M., Martin-Vasallo P. (2002). Glucose transport and metabolism in chondrocytes: A key to understanding chondrogenesis, skeletal development and cartilage degradation in osteoarthritis. Histol. Histopathol..

[145] Mobasheri A. (2012). Glucose: An energy currency and structural precursor in articular cartilage and bone with emerging roles as an extracellular signaling molecule and metabolic regulator. Front. Endocrinol..

[146] Stockwell R.A., Harrison R.J., McMinn R.M. (1979). Chapter 5. Chondrocyte nutrition, cartilage boundaries and permeability. Biology of Cartilage Cells.

[147] Armstrong C.G., Gardner D.L. (1977). Thickness and distribution of human femoral head articular cartilage. Changes with age. Ann. Rheum. Dis..

[148] Pedley T.J. (1983). Calculation of unstirred layer thickness in membrane transport experiments: A survey. Q. Rev. Biophys..

[149] Korjamo T., Heikkinen A.T., Monkkonen J. (2009). Analysis of unstirred water layer in in vitro permeability experiments. J. Pharm. Sci..

[150] Crank J., Crank E.P.J. (1975). The Mathematics of Diffusion.

[151] Srinivasan B. (2022). A guide to the Michaelis–Menten equation: Steady state and beyond. FEBS J..

[152] Torzilli P.A., Adams T.C., Mis R.J. (1987). Transient solute diffusion in articular cartilage. J. Biomech..

[153] Pouran B., Arbabi V., Weinans H., Zadpoor A.A. (2016). Application of multiphysics models to efficient design of experiments of solute transport across articular cartilage. Comput. Biol. Med..

[154] Wallis W.J., Simkin P.A., Nelp W.B. (1987). Protein traffic in human synovial effusions. Arthritis Rheum..

[155] Hwang H.S., Kim H.A. (2015). Chondrocyte apoptosis in the pathogenesis of osteoarthritis. Int. J. Mol. Sci..

[156] Maroudas A. (1968). Physicochemical properties of cartilage in the light of ion exchange theory. Biophys. J..

[157] Dean D., Seog J., Ortiz C., Grodzinsky A.J. (2003). Molecular-level theoretical model for electrostatic interactions within polyelectrolyte brushes: Applications to charged glycosaminoglycans. Langmuir.

[158] Karsdal M.A., Madsen S.H., Christiansen C., Henriksen K., Fosang A.J., Sondergaard B.C. (2008). Cartilage degradation is fully reversible in the presence of aggrecanase but not matrix metalloproteinase activity. Arthritis Res. Ther..

[159] Kumar L., Bisen M., Khan A., Kumar P., Patel S.K.S. (2022). Role of matrix metalloproteinases in musculoskeletal diseases. Biomedicines.

[160] Newman A.P. (1998). Articular cartilage repair. Am. J. Sports Med..

[161] Bay-Jensen A.C., Hoegh-Madsen S., Dam E., Henriksen K., Sondergaard B.C., Pastoureau P., Qvist P., Karsdal M.A. (2010). Which elements are involved in reversible and irreversible cartilage degradation in osteoarthritis?. Rheumatol. Int..

[162] Heinemeier K.M., Schjerling P., Heinemeier J., Møller M.B., Krogsgaard M.R., Grum-Schwensen T., Petersen M.M., Kjaer M. (2016). Radiocarbon dating reveals minimal collagen turnover in both healthy and osteoarthritic human cartilage. Sci. Transl. Med..

[163] Bank R.A., Bayliss M.T., Lafeber F.P., Maroudas A., Tekoppele J.M. (1998). Ageing and zonal variation in post-translational modification of collagen in normal human articular cartilage. The age-related increase in non-enzymatic glycation affects biomechanical properties of cartilage. Biochem. J..

[164] Simkin P.A. (2013). Assessing biomarkers in synovial fluid: Consider the kinetics of clearance. Osteoarthr. Cartil..

[165] Hunter D.J., Nevitt M., Losina E., Kraus V. (2014). Biomarkers for osteoarthritis: Current position and steps towards further validation. Best. Pract. Res. Clin. Rheumatol..

[166] Van Rossom S., Smith C.R., Zevenbergen L., Thelen D.G., Vanwanseele B., Van Assche D., Jonkers I. (2017). Knee cartilage thickness T1 and T2 relaxation time are related to articular cartilage loading in healthy adults. PLoS ONE.

[167] Roemer F.W., Guermazi A., Demehri S., Wirth W., Kijowski R. (2022). Imaging in osteoarthritis. Osteoarthr. Cartil..

[168] Tiderius C.J., Olsson L.E., Leander P., Ekberg O., Dahlberg L. (2003). Delayed gadolinium-enhanced MRI of cartilage (dGEMRIC) in early knee osteoarthritis. Magn. Reson. Med..

[169] del Río E., Vergés J. (2024). Exploring the influence of physical activity on the efficacy of chondroprotective agents for osteoarthritis: The role of diffusion conditions. Med. Hypotheses.

[170] Fernández-Martín S., González-Cantalapiedra A., Muñoz F., García-González M., Permuy M., López-Peña M. (2021). Glucosamine and chondroitin sulfate: Is there any scientific evidence for their effectiveness as disease-modifying drugs in knee osteoarthritis preclinical studies? A systematic review from 2000 to 2021. Animals.

[171] del Río E. (2025). Rethinking osteoarthritis management: Synergistic effects of chronoexercise, circadian rhythm, and chondroprotective agents. Biomedicines.

[172] Moriyama H., Yoshimura O., Kawamata S., Takayanagi K., Kurose T., Kubota A., Hosoda M., Tobimatsu Y. (2008). Alteration in articular cartilage of rat knee joints after spinal cord injury. Osteoarthr. Cartil..

[173] Lories R.J., Luyten F.P. (2011). The bone–cartilage unit in osteoarthritis. Nat. Rev. Rheumatol..

[174] Goldring S.R., Goldring M.B. (2016). Changes in the osteochondral unit during osteoarthritis: Structure, function and cartilage-bone crosstalk. Nat. Rev. Rheumatol..

[175] Suri P., Morgenroth D.C., Hunter D.J. (2012). Epidemiology of osteoarthritis and associated comorbidities. PM&R.

[176] Grønne D.T., Roos E.M., Ibsen R., Kjellberg J., Skou S.T. (2021). Cost-effectiveness of an 8-week supervised education and exercise therapy programme for knee and hip osteoarthritis: A pre-post analysis of 16 255 patients participating in Good Life with osteoArthritis in Denmark (GLA:D). BMJ Open.

[177] Hoffmann G.R. (2009). A perspective on the scientific, philosophical, and policy dimensions of hormesis. Dose Response.

[178] Ingram K.R., Wann A.K.T., Angel C.K., Coleman P.J., Levick J.R. (2008). Cyclic movement stimulates hyaluronan secretion into the synovial cavity of rabbit joints. J. Physiol..

[179] Stokols D. (1996). Translating social ecological theory into guidelines for community health promotion. Am. J. Health Promot..

[180] Frieden T.R. (2010). A framework for public health action: The health impact pyramid. Am. J. Public Health.

[181] Luong M.-L.N., Cleveland R.J., Nyrop K.A., Callahan L.F. (2012). Social determinants and osteoarthritis outcomes. Aging Health.

[182] Speziali A., Delcogliano M., Tei M., Placella G., Chillemi M., Tiribuzi R., Cerulli G. (2015). Chondropenia: Current concept review. Musculoskelet. Surg..

[183] Lee D.A., Bader D.L. (1997). Compressive strains at physiological frequencies influence the metabolism of chondrocytes seeded in agarose. J. Orthop. Res..

[184] Grumbles R.M., Howell D.S., Howard G.A., Roos B.A., Setton L.A., Mow V.C., Ratcliffe A., Muller F.J., Altman R.D. (1995). Cartilage metalloproteases in disuse atrophy. J. Rheumatol. Suppl..

[185] Hambly K., Bobic V., Wondrasch B., Van Assche D., Marlovits S. (2006). Autologous chondrocyte implantation postoperative care and rehabilitation: Science and practice. Am. J. Sports Med..

[186] McAdams T.R., Mithoefer K., Scopp J.M., Mandelbaum B.R. (2010). Articular cartilage injury in athletes. Cartilage.

[187] Kong L., Wang L., Meng F., Cao J., Shen Y. (2017). Association between smoking and risk of knee osteoarthritis: A systematic review and meta-analysis. Osteoarthr. Cartil..

[188] Jaleel A., Golightly Y.M., Alvarez C., Renner J.B., Nelson A.E. (2021). Incidence and progression of ankle osteoarthritis: The johnston county osteoarthritis project. Semin. Arthritis Rheum..

